# Supplementary material for: Mechanochemistry-driven engineering of 0D/3D heterostructure for designing highly luminescent Cs–Pb–Br perovskites
Source: Nat Commun. 2022 Jul 23;13:4263. doi: 10.1038/s41467-022-31924-x (PMC9308791; doi:10.1038/s41467-022-31924-x)
Supplement: Supplementary file 1 — Supplementary Information [file 41467_2022_31924_MOESM1_ESM.pdf]

Supplementary Information

Mechanochemistry-driven engineering of 0D/3D heterostructure for designing highly luminescent Cs–Pb–Br perovskites

Baek, Lee *et al.*

Supplementary Note 1: Time evolution of optical properties during MCS process

1.1 Low-energy versus high-energy ball milling

We primarily focused on selecting the appropriate mechanochemical synthesis (MCS) method to perform systematic evaluation of the intermediate samples. Among various MCS methods (e.g., hand-grinding, rotating mill-type, planetary-type, and shaker-type ball mill), the hand-grinding method was excluded due to practicality issues. Next, methods that can apply uniform forces to the precursors throughout the entire process was largely classified into low-energy and high-energy ball milling. Rotating mill-type (Supplementary Fig. 1a) and shaker-type (Supplementary Fig. 1b) ball mill represent low-energy and high-energy ball mill, respectively. Since post-synthetic structural transformation between Cs–Pb–Br perovskite phases have been reported to occur easily when temperature is elevated from room temperature[1–3], it is important to maintain a constant temperature during the synthesis process in order to thoroughly evaluate the intermediate samples. Otherwise, it becomes difficult to disentangle the effect caused by elevated temperature from the effect caused by the mechanical energy. In the case of shaker-type ball mill (i.e., high-energy ball mill), mechanochemical reaction occurs rather aggressively and the temperature of the synthesis environment rises up to ~70°C according to our experimental setup. On the other hand, in the case of rotating mill-type ball mill (i.e., low-energy ball mill), the temperature is maintained constant at room temperature of ~25°C. Temperature measurements are conducted through infrared thermometer (DK400L1, Daekwang Inc.). The temperature evolution of each setup is presented in Supplementary Fig. 1c. Therefore, among the numerous methods available for MCS, the rotating mill-type ball milling is the most appropriate method for observing evolutional properties with high reliability.

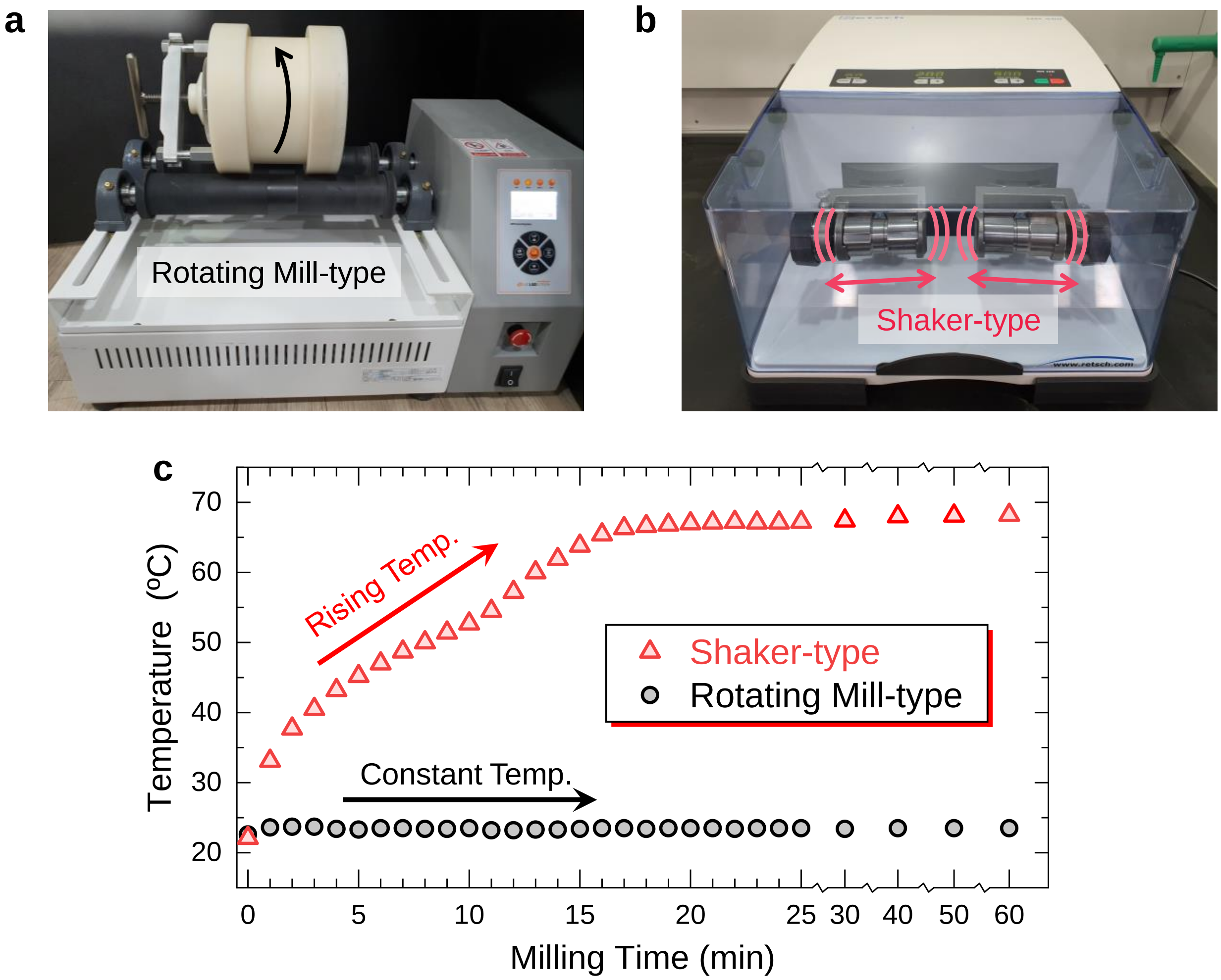

Supplementary Fig. 1 | Rotating mill-type and shaker-type ball mill. a,b, Experimental setup images of rotating mill-type (**a**) and shaker-type ball mill (**b**). **c,** Evolution of temperature in each condition. The rotating mill-type ball mill was carried out in the equal environment as indicated in the Methods section and the shaker-type ball mill was performed with MM-400 straight ball-mill from Retsch Inc., at a frequency of 30 Hz with two stainless-steel balls of radius 1.9 cm.

1.2 Pure perovskite phases acquired through MCS

Phase-pure products of three-dimensional (3D) $CsPbBr_3$, zero-dimensional (0D) Cs_4PbBr_6, and two-dimensional (2D) $CsPb_2Br_5$ were obtained through the rotating mill method. The initial stoichiometry of the precursors were varied according to the respective product ($CsBr:PbBr_2$ = 1:1, 4:1, and 1:2 for $CsPbBr_3$, Cs_4PbBr_6, $CsPb_2Br_5$, respectively); the powder X-ray diffraction (PXRD) patterns are presented in Supplementary Fig. 2. Combining these results with the discussion from Supplementary Note 1.1, rotating mill-type ball milling not only maintains the temperature at a constant level, but also proves that it can provide sufficient energy to synthesize all three phases.

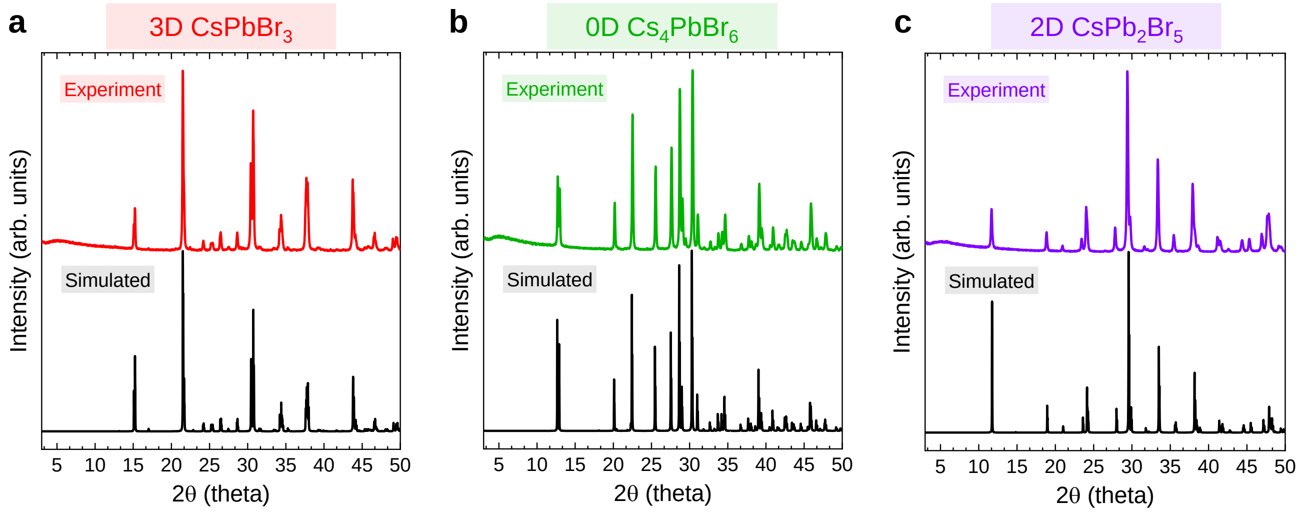

Supplementary Fig. 2 | Mechanochemically synthesized Cs–Pb–Br perovskites. a–c, Measured (top) and simulated (bottom) PXRD patterns of 3D $CsPbBr_3$ (**a**), 0D Cs_4PbBr_6 (**b**), and 2D $CsPb_2Br_5$ (**c**). Simulated results of 3D $CsPbBr_3$ and 0D Cs_4PbBr_6 are from COD ID 4510745 (ref. [4]) and 4002857 (ref. [5]), respectively, and 2D $CsPb_2Br_5$ is from ref. [6].

1.3 PLQE measurement of powder samples

When calculating the photoluminescence (PL) quantum efficiency (QE) values for powder samples, we followed the method reported by de Mello et al.[7]. Here, we note that the correction term acquired from the indirect configuration needs to be applied when calculating the PLQE value for high scattering samples (e.g., solid-state samples). Schematic illustrations of direct configuration and indirect configuration in the PLQE measurement process are shown in Supplementary Figs. 3a and 3b, respectively. Photos of the inner structure of the integrating sphere in each configuration are presented in Supplementary Figs. 3c and 3d. In the direct configuration (Supplementary Figs. 3a and 3c), the excitation light directly irradiates the sample, whereas in the indirect configuration (Supplementary Figs. 3b and 3d), sample is removed from the incident light path. Initial PLQE values (before applying any correction) are acquired through direct configuration. However, when measuring samples with high scattering properties, considerable amount of incident light is diffused back to the inner surface of integrating sphere, thereby requiring the emission and absorption by indirect excitation to be considered in calculating the accurate PLQE value and this process is termed as indirect excitation correction. Here, indirect excitation is defined as an excitation of the sample when the incident light is (i) scattered from the sample, (ii) diffused in the integrating sphere, and (iii) re-excites the sample. Eq. 1 is the formula of PLQE correction when the indirect excitation is taken into account

$$\varepsilon_{in}' = \frac{E_2 - \frac{L_2}{L_3}E_3}{L_1 - \frac{L_1}{L_3}L_2} \approx \frac{E_2 - \frac{L_2}{L_3}E_3}{L_1 - L_2} = \varepsilon_{in} - \frac{\frac{L_2}{L_3}E_3}{L_1 - L_2} = \varepsilon_{in} - \Delta\varepsilon_{corr} \qquad \text{(Eq. 1)}$$

where L_1 is the power of excitation light scattered by the inner surface of the integrating sphere before loading the sample and L_2, L_3, E_2, and E_3 are as defined in Supplementary Figs. 3a and 3b. Here, a portion of the emission power which is induced through indirect excitation (L_2E_3/L_3) needs to be subtracted from the power of the incident emission by direct excitation (E_2). Considering that L_1 and L_3 are generally similar values, the PLQE value after correction (ε_{in}') is always smaller than the PLQE value before

correction (ε_{in}) by the difference of $\Delta\varepsilon_{corr}$ which is a positive value. Hence, not taking the indirect excitation correction into account results in overestimating the PLQE values. In our case for the 360-min sample, PL spectra measured in the direct configuration and indirect configuration along with the plain excitation light are shown in Supplementary Fig. 4. PLQE value of 10.7 % reported in Fig. 2b of the main text is the corrected value after taking the indirect excitation into account. Before the correction, our PLQE value was calculated to be 13.7 % where we can identify that the correction process reduces the PLQE value by 3.0 %p. Note that the corrected PLQE value of 10.7 % is an accurate calculation before applying the approximation in Eq. 1. The process of approximating L_3 to L_1 and the following $\Delta\varepsilon_{corr}$ term was introduced to explain that the correction generally decreases the PLQE value. In summary, all the PLQE values shown in Fig. 2b are the results of indirect excitation correction and not considering such process might result in overestimating the PLQE values.

In addition, we note that the relatively low PLQE values of our powder samples compared to those with literature[5,8,9] stems from the different synthesis approach in terms of sample washing and synthesis methods. In particular, many reports present PLQE values for the solid samples after washing the as-formed product with dimethyl sulfoxide (DMSO). For instance, PLQE value of 45 % was achieved only after washing the sample with DMSO[5,8] whereas only ~7% PLQE was achieved before the washing process[8]. Hence, our PLQE values for the powder samples are comparable with reference values for the samples in their as-synthesized form (i.e., before any washing process). Furthermore, depending on the amount of DMSO used for washing, PLQE values can vary within a large range of 60–95% depending on the amount of DMSO used[9]. However, the PLQE data in Fig. 2b of the main text were measured in as-synthesized forms since our main focus was to track the time evolution of intrinsic properties of powder samples induced only through mechanochemical reactions (i.e., no intermixing effects due to the DMSO washing).

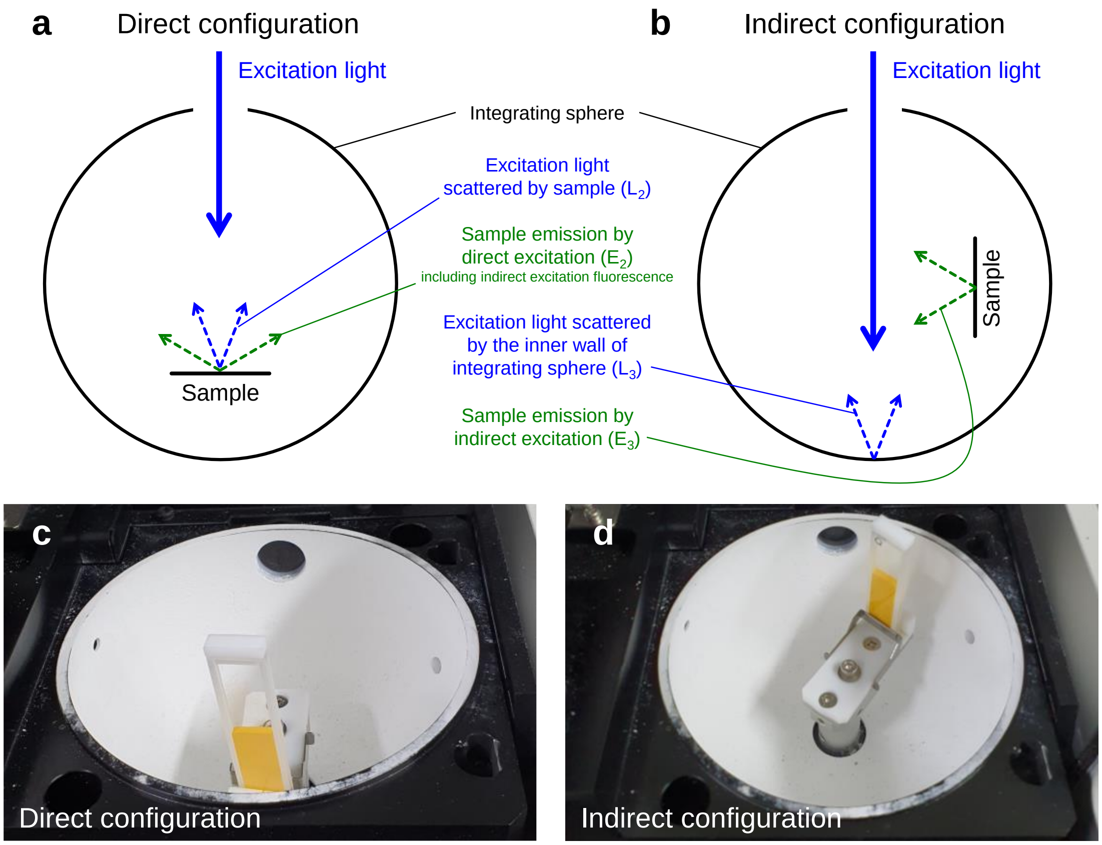

Supplementary Fig. 3 | Direct and indirect configuration. a,b, Schematic illustration of direct configuration (**a**) and indirect configuration (**b**). **c,d,** Experimental setup images showing the inner structure of the integrating sphere in direct configuration (**c**) and indirect configuration (**d**). There are three circle-shaped ports inside the integrating sphere as can be seen in **c** and **d**. The circle on the top is the excitation light inlet port and smaller circles on the left and right is the emission light outlet port and the calibrated light source port, respectively.

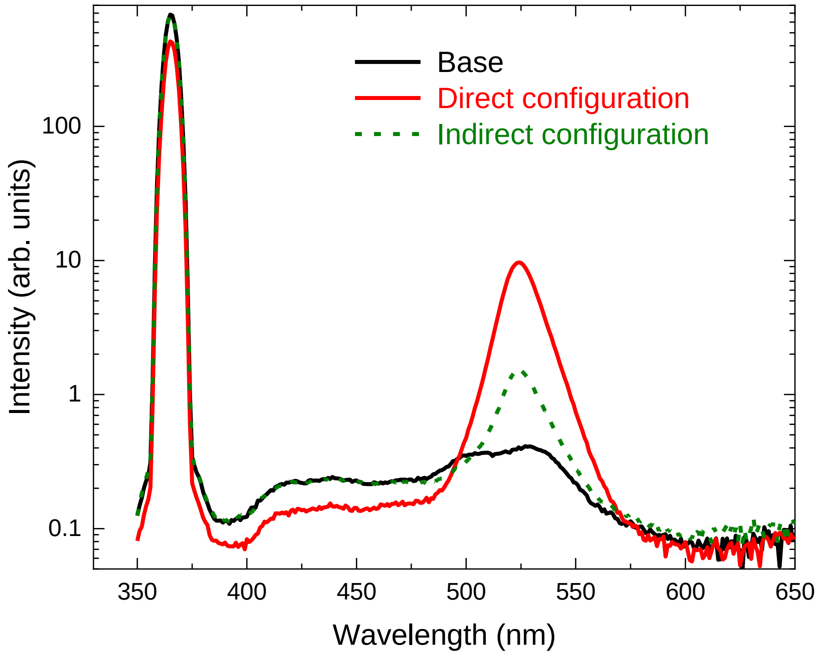

Supplementary Fig. 4 | PLQE correction. PL spectra measured in the direct configuration (solid red line) and in the indirect configuration (short dashed green line) of the 180-min sample along with the plain excitation light denoted as 'Base' with a peak wavelength of 365 nm (solid black line). Note that the PL intensity is presented in logarithmic scale.

1.4 Time evolution of emissive properties

There is no green-colored emissive species present in the 0-min sample which is shown as an almost flat baseline compared to 5-min sample in Supplementary Fig. 5a. Here, the PL spectrum of 5-min sample is shown for comparison and it is the same data from Fig. 2c of the main text only before normalization. Supplementary Fig. 5b shows the absorption spectrum of the 0-min sample. The spectrum of 5-min sample here is also shown for comparison and it is the same data from Fig. 2d of the main text. Although a slight degree of 3D $CsPbBr_3$ formation was confirmed in the 0-min sample from the band-to-band absorption with an onset of ~532 nm, this is likely to represent some unintended formation of the 3D phase despite our careful preparation of the sample by a minimal mixing of the precursors (CsBr and $PbBr_2$) for evenly distributing inside the sample holding cell for measurement. Even without the presence of the stainless steel balls, mechanochemical reaction can occur through the collision of the precursor powder particles. However, the corresponding PLQE value of the 0-min sample is 0.07 % (Supplementary Fig. 5c), thus conclusively showing the absence of any significant emission source in the sample before milling.

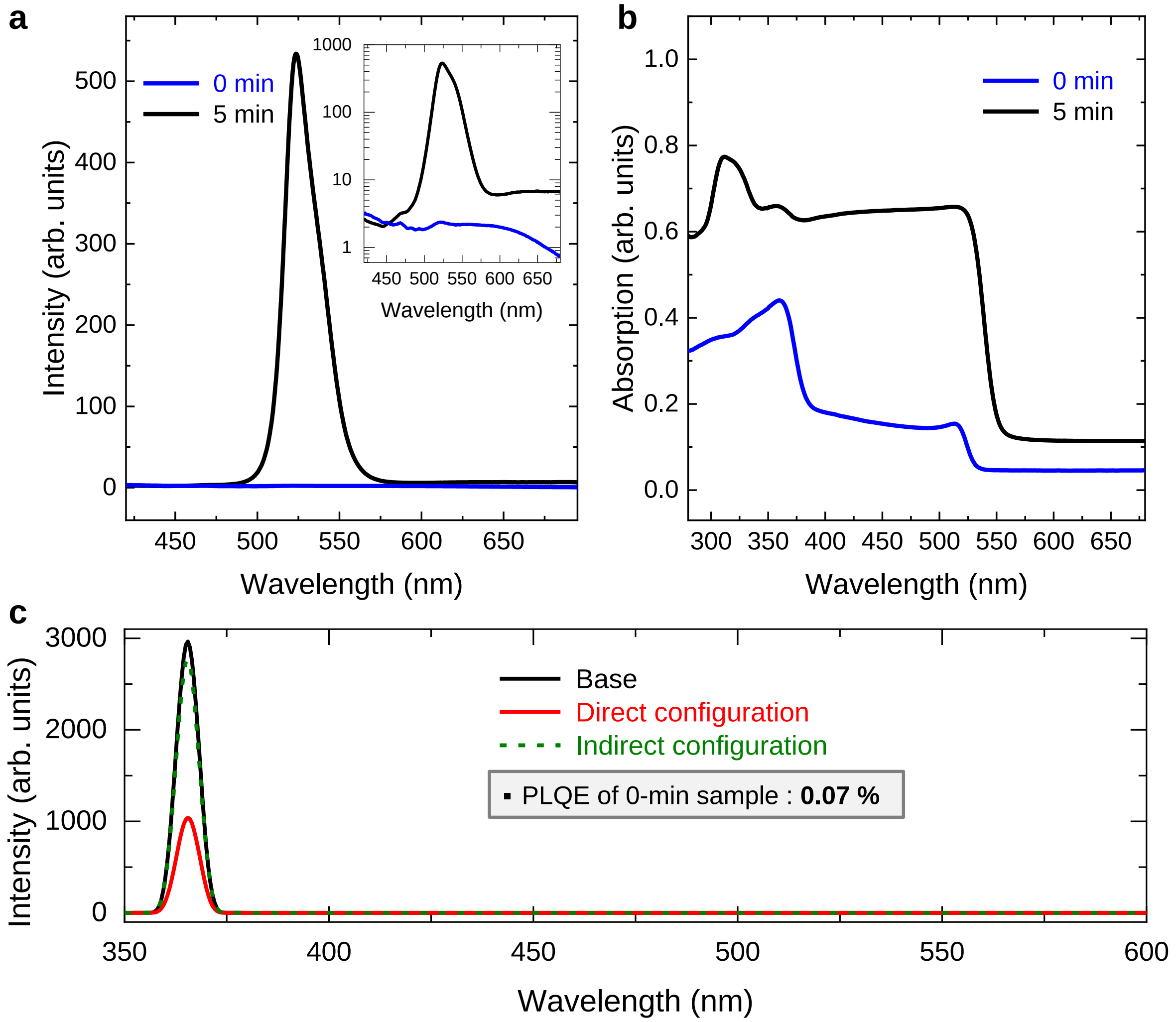

Supplementary Fig. 5 | Absence of emission in the 0-min sample. a, PL spectra of 0-min and 5-min sample with an excitation wavelength of 365 nm. Inset is the same spectra in logarithmic scale. **b,** Absorption spectra of 0-min and 5-min sample. **c,** PL spectra of the 0-min sample for PLQE measurement with an excitation wavelength of 365 nm.

Supplementary Figure 6 shows the time evolution of photoluminescence peak position and full width at half maximum (FWHM) of the corresponding peak in the synthesis process of 0D Cs_4PbBr_6 (through CsBr and $PbBr_2$ precursors mixed in a stoichiometric ratio of 4:1). We note that the PL peak gradually blue-shifts from ~180 min of milling time until the completion of synthesis. In the meantime, the FWHM slowly decreases until ~660 min of milling time is reached. The final sample (i.e., 2880-min sample) shows a large change in both properties, i.e., PL peak position blue-shifts by ~13 nm and FWHM increases by ~9 nm, compared to the samples in the previous stage (until the 1320-min sample).

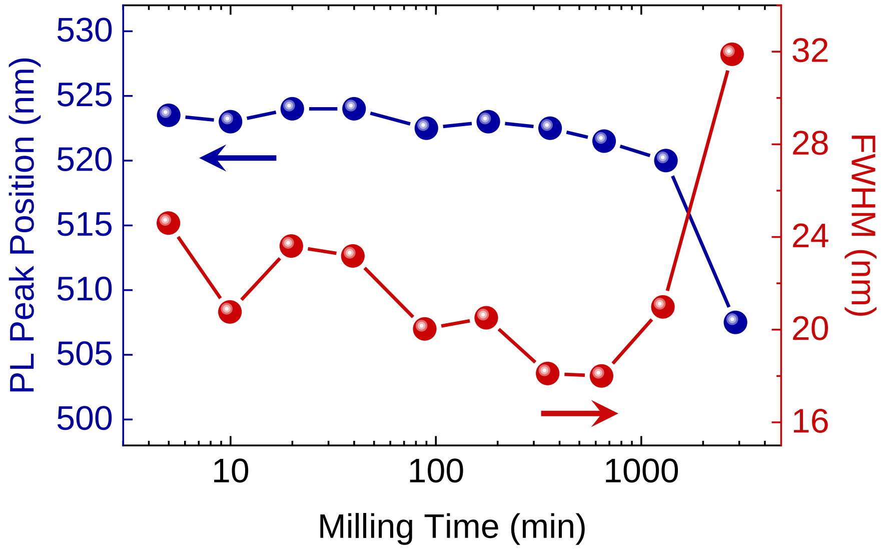

Supplementary Fig. 6 | Evolution of PL properties. Evolution of (blue) PL peak position and (red) FWHM with successive increase in milling time. These parameters are obtained from the PL spectra in Fig. 2c of the main text.

Properties of 3D $CsPbBr_3$ and 0D Cs_4PbBr_6 in the synthesized samples can be examined through controlling the excitation wavelength of the PL spectrum and monitoring different emission wavelength of the PLE spectrum. Note that the PL spectra in Fig. 2c from the main text were measured at an excitation wavelength of 365 nm. When the PL excitation (PLE) spectrum (i.e., PL spectrum from the sample with varying excitation wavelength) was monitored at 523-nm emission for 180-min and 2880-min samples (i.e., the brightest and the synthesis-completed sample, respectively), two peaks at 294 and 365 nm were observed with the 365-nm peak being higher in intensity (Supplementary Fig. 7a). This confirms that the excitation source of 365-nm wavelength in Figs. 2a and 2c from the main text was suitable for observing the emission characteristics of 523 nm. More importantly, the "hole-burning" region was found in between the 294 and 365 nm regions (marked as blue arrow in Supplementary Fig. 7a), which is in accordance with refs. [9–11]. In other words, when the samples were illuminated with an excitation wavelength of 310 nm (i.e., located inside the "hole-burning" region), emission at 523 nm was not observed (Supplementary Fig. 7b); instead, emission at 375 nm of wavelength was measured and the intensity of the 2880-min sample was much greater than that of the 180-min sample. This broad (FWHM of ~60 nm) ultraviolet (UV) emission corresponds to the intrinsic emission from 0D Cs_4PbBr_6 (refs. [12–15]). More specifically, this emission is attributed to the radiative decay of a Frenkel exciton at Pb^{2+} site in isolated $[PbBr_6]^{4-}$ octahedra in the 0D phase. When the PLE spectrum was monitored at 375 nm for the 2880-min sample, it showed a similar tendency with the absorption spectra from Fig. 2d of the main text (see the similarity between black dashed line and green solid line in Supplementary Fig. 7c). The overlap with PLE spectrum monitored at 523 nm emission (red dashed line in Supplementary Fig. 7c; same spectrum as in Supplementary Fig. 7a) shows that the peaks from PLE spectrum monitored at 375 nm emission (black dashed line) and absorption spectrum (green solid line) coincides with the aforementioned "hole-burning" region. This refers that the desired green emission at 523 nm is not observed within the excitation

wavelength range that 0D Cs_4PbBr_6 can absorb (in other words, emission from 3D $CsPbBr_3$ is active when absorption from 0D Cs_4PbBr_6 is inactive). Finally, upon excitation at 294 nm (i.e., another peak from Supplementary Fig. 7a), the absorption from 0D Cs_4PbBr_6 is inactive (note that 294 nm locates at the local minimum of 0D absorption from Supplementary Fig. 7c) such that the green emission at 523 nm with narrow FWHM of 18 nm can be observed, similar to the excitation at 365 nm. All these results imply that 3D $CsPbBr_3$ and 0D Cs_4PbBr_6 are not only intermixed in the samples but also possess interface between phase moieties, because energy transfer in different excitation wavelength is observed to be directly influenced by the properties of each phase.

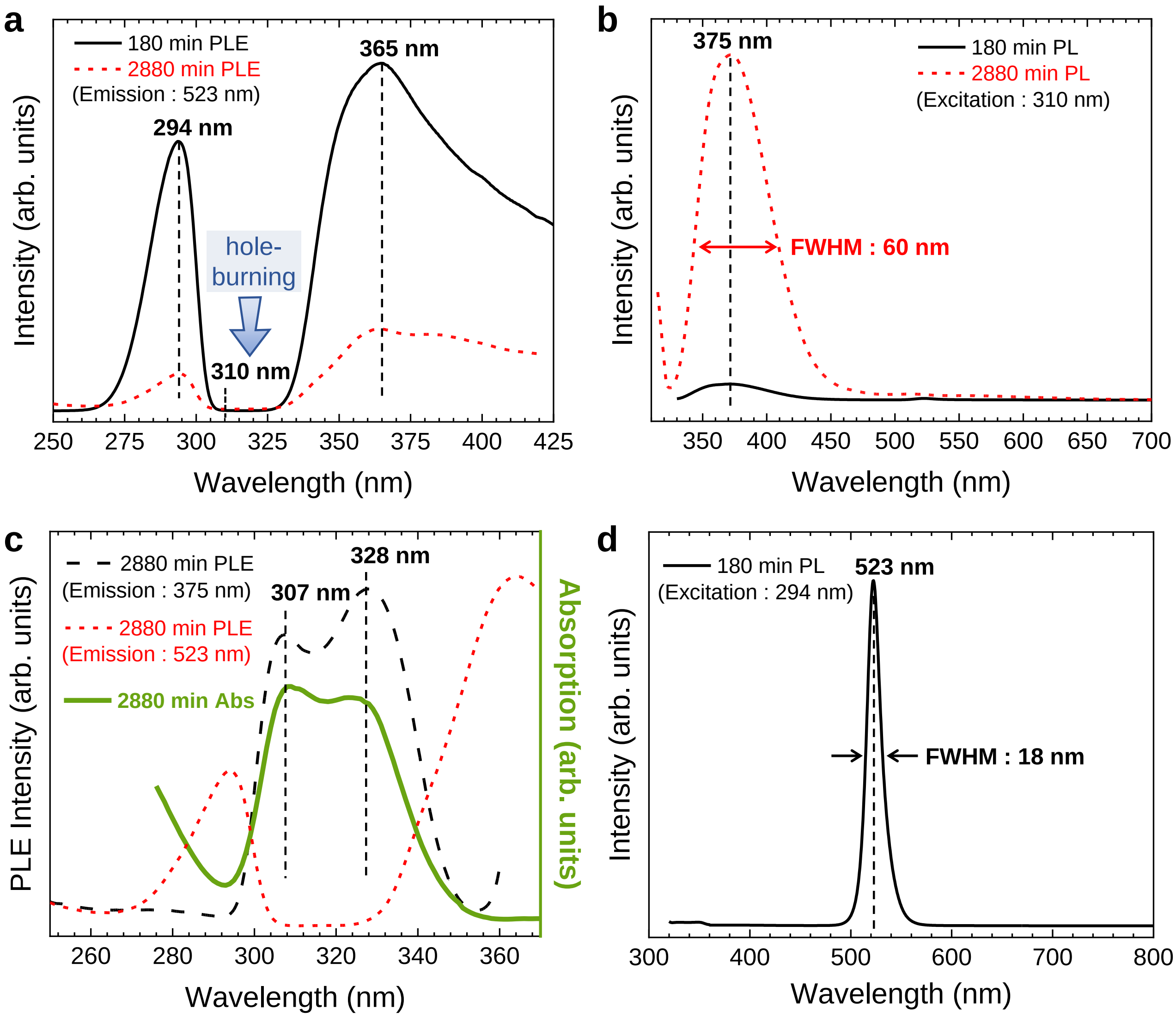

Supplementary Fig. 7 | PL and PLE spectra of intermediate and final samples. a, PLE spectra of 180-min and 2880-min samples monitored at emission wavelength of 523 nm. **b,** PL spectra of 1880-min and 2880-min samples with excitation wavelength of 310 nm. **c,** PLE spectra of 2880-min sample monitored at emission wavelength of (black) 375 nm and (red) 523 nm, and (green) a section of the absorption spectrum for the 2880-min sample from Fig. 2d of the main text. **d,** PL spectrum of 180-min sample with excitation wavelength of 294 nm.

1.5 Time evolution of optical bandgaps and absorption coefficients

Supplementary Figure 8 is the Tauc plot drawn near the onset position of 550 nm (~2.3 eV) which is obtained from the absorption spectra in Fig. 2d of the main text.

Supplementary Figure 9a shows the evolution of ratio between the absorption and scattering constants (α). α can be interpreted as having the identical physical meaning as the absorption coefficient in the Tauc plot. α of 3D $CsPbBr_3$ (red diamond symbol in Supplementary Fig. 9a) is obtained from an incident wavelength of 517 nm (i.e., 2.4 eV; bandgap of $CsPbBr_3$). It can be seen that the level of 3D $CsPbBr_3$ absorption is high in the initial stage of the synthesis (~40 min of milling time) and decreases as the synthesis proceeds to the final stage. This indicates that the content of 3D $CsPbBr_3$ is high in the mixture up to the middle stage of the synthesis, and then decreases toward the latter stage. α of 0D Cs_4PbBr_6 (black triangle symbol in Supplementary Fig. 9a) is obtained from an incident wavelength of 318 nm (i.e., 3.9 eV; bandgap of Cs_4PbBr_6). It can be seen that the degree of 0D Cs_4PbBr_6 absorption increases as the synthesis proceeds to the final stage. This indicates the formation of 0D Cs_4PbBr_6 from the initial stage to the completion of synthesis.

Supplementary Figure 9b shows a spectrum of α with respect to energy near the bandgap of 0D Cs_4PbBr_6 (3.9 eV). In accordance with Supplementary Fig. 9a, the degree of 0D Cs_4PbBr_6 absorption near its bandgap increases as the synthesis proceeds. This localized absorption is due to spin–orbit allowed transitions of the $[PbBr_6]^{4-}$ octahedra in the lattice of 0D Cs_4PbBr_6 (ref. [12,13,16,17]). We can also observe a noticeable splitting of peaks around 3.9 eV as the synthesis proceeds. The doublet pattern of this band (also named as the “A band”) is attributed to the Jahn–Teller effect on the degenerate electronic states of Pb^{2+} ions[18].

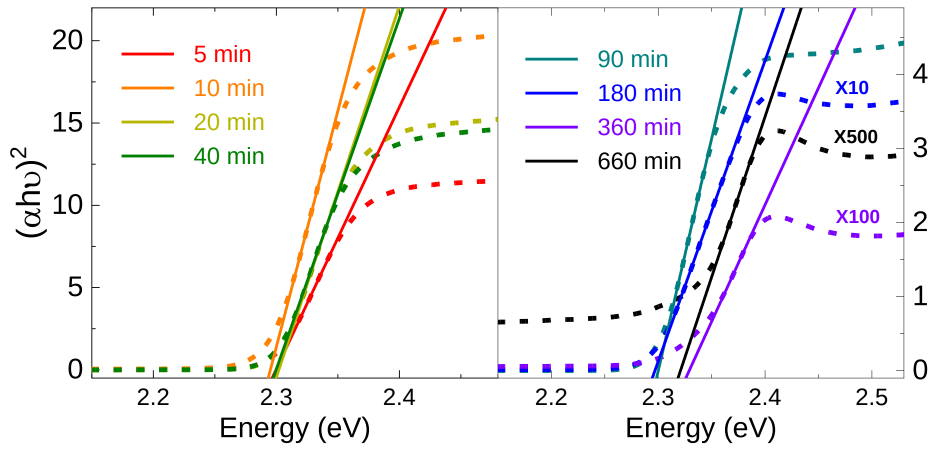

Supplementary Fig. 8 | Nearly constant optical bandgap. Tauc plot near the onset position of 550 nm (~2.3 eV) obtained from the absorption spectra in Fig. 2d of the main text. The spectra of 180, 360, and 660-min samples are magnified by 10, 100, and 500 times, respectively to clearly display the onset point.

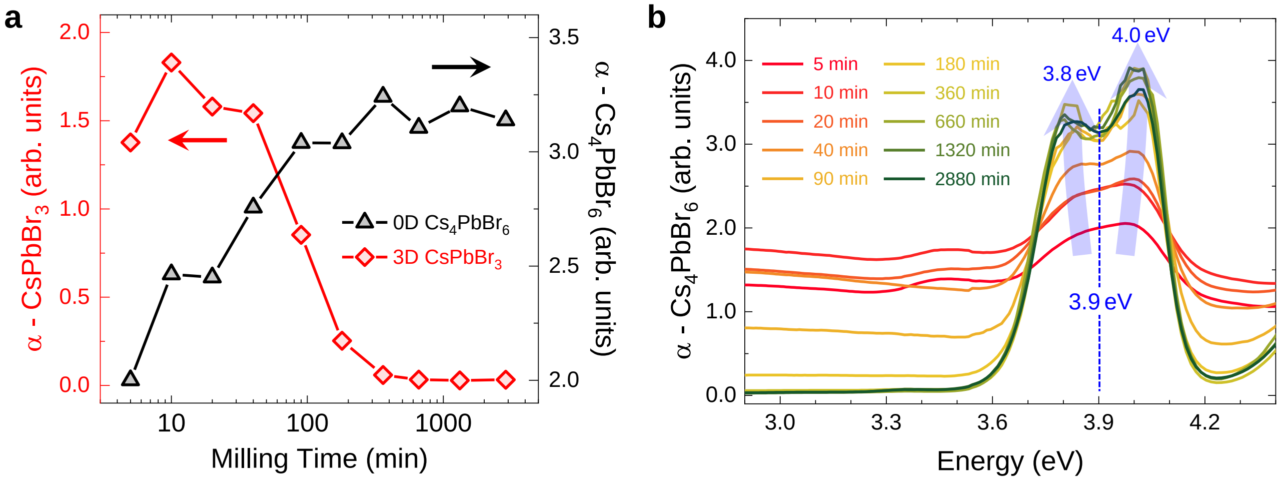

Supplementary Fig. 9 | Spectral evolution of α. a, Time evolution of α in (red diamonds) 3D $CsPbBr_3$ from excitation wavelength of 517 nm and (black triangles) 0D Cs_4PbBr_6 from excitation wavelength of 318 nm. **b,** Time evolution of α near 3.9 eV, i.e., the bandgap of 0D Cs_4PbBr_6.

Supplementary Note 2: Spatially identifying green emissive regions

2.1 Synthesized powder samples observed through optical microscope

Synthesized powder samples with different milling times are observed through optical microscope as shown in Supplementary Fig. 10. Similar to the trend observed in the top image of Fig. 2a from the main text, yellow portion of the powder decreases whereas the white portion increases. Since the colors in 90-min sample are evenly mixed, it is expected to have the most diverse types of components, which justifies the selection of the 90-min sample for the examination in Fig. 2f from the main text.

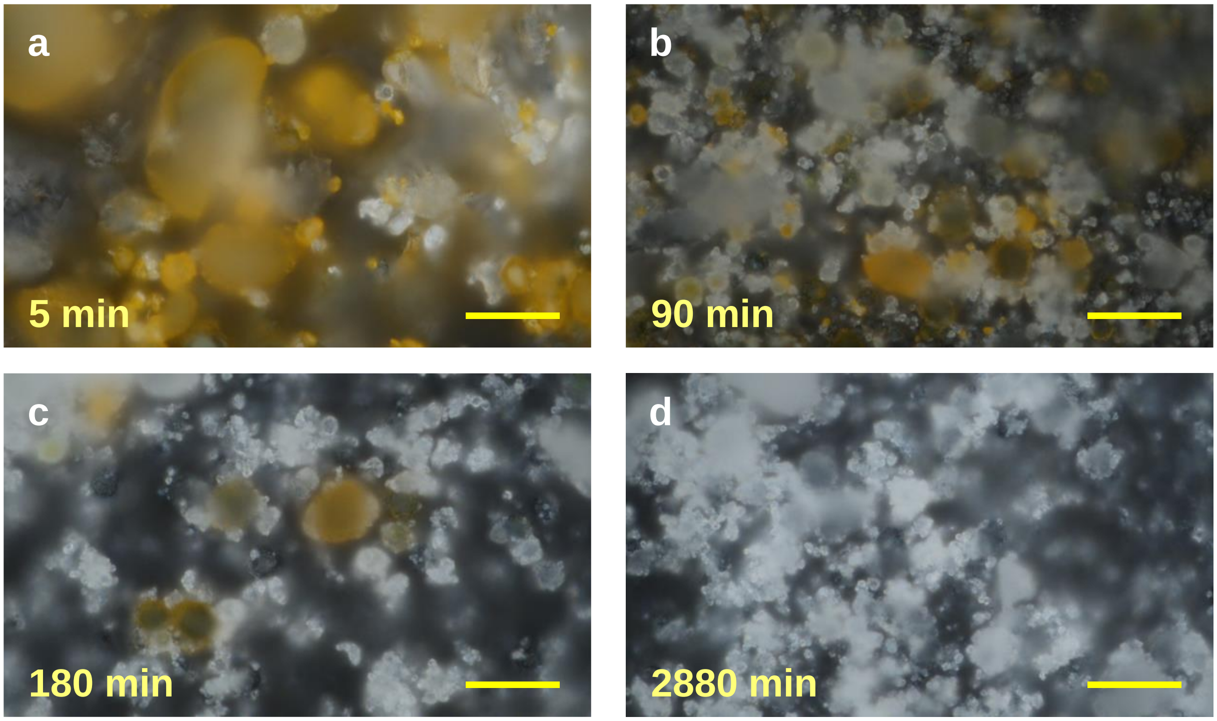

Supplementary Fig. 10 | Optical microscope images. a–d, Images of synthesized powder samples with 5 min (**a**), 90 min (**b**), 180 min (**c**), and 2880 min (**d**) of milling times. All scale bars, 20 µm.

2.2 Microscopic examination of bright green regions in the synthesized powder

In order to ascribe the observed green emission to the presence of the 3D $CsPbBr_3$ phase, a prepared powder sample was examined through optical microscopy with complementary confocal PL imaging. Numerous green fluorescence regions were clearly observed in the 90-min sample (see the left image of Supplementary Fig. 11a), which had the most diverse range of distinguishable Cs–Pb–Br polymorphs (see Supplementary Fig. 10 for detailed comparisons) and was best suited for identifying the local origin of the bright emission. When confocal PL mapping was performed in the same region as the optical image (right image of Supplementary Fig. 11a), the highly luminescent regions could be clearly identified; they coincided with the green regions in the optical image (see circled and squared regions in Supplementary Fig. 11a). A detailed mapping of integrated PL intensity for the bright regions (marked by yellow squares in Supplementary Fig. 11a) was performed and showed that the PL intensity increased toward the centers of the emitters (two green areas in Supplementary Fig. 11b), where a narrow FWHM of the emission peak (15.9 nm) was observed (inset of Supplementary Fig. 11b). When mapping the PL peak wavelength of the same region (Supplementary Fig. 11c), a red-shift of 6–9 nm was observed when approaching the emission center. Because the PL emission blue-shifted as the synthesis progressed (Supplementary Fig. 6), we can infer that the emission center contains relatively higher portions of unreacted phases than the exterior area. The gradual red-shift from the surface to the interior of the reacting particles in Supplementary Fig. 11c suggests an inward directionality of the MCS reaction (i.e., endotaxial synthesis), which becomes a crucial factor when discussing Fig. 5 of the main text and Supplementary Note 5.

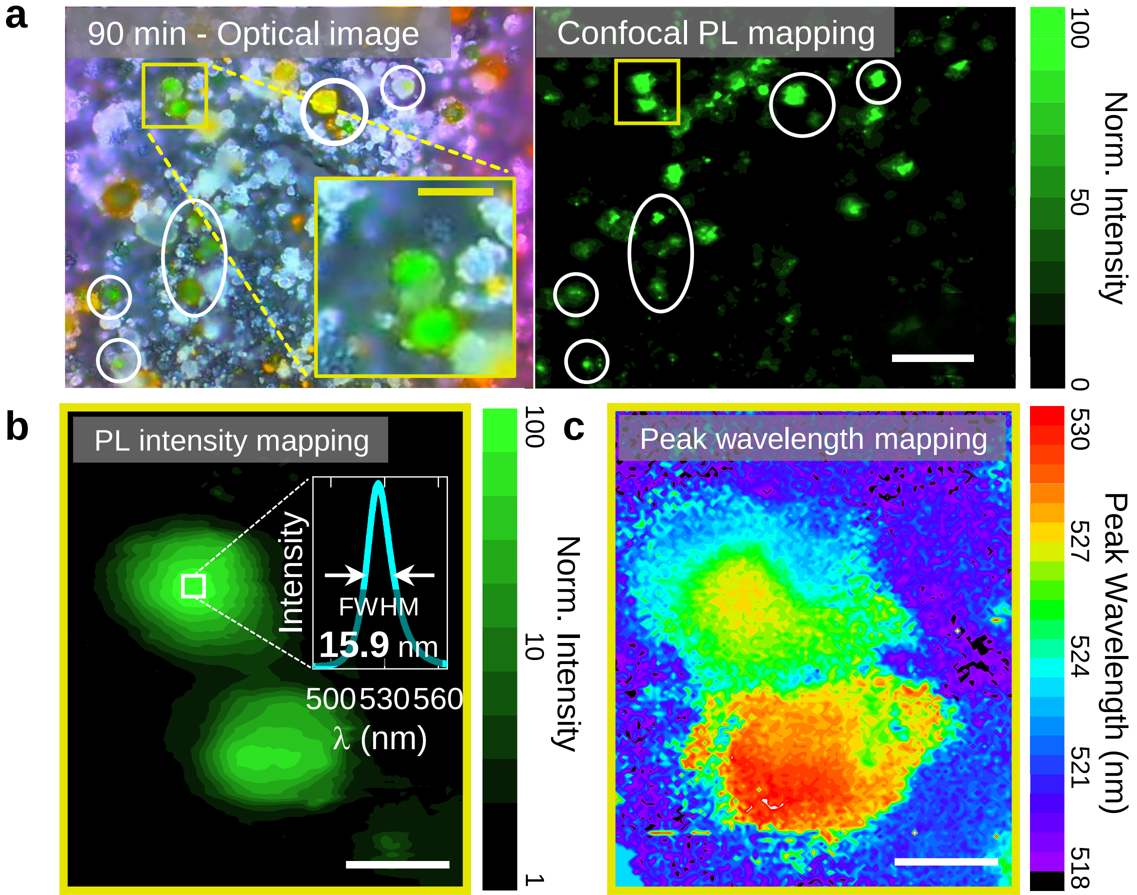

Supplementary Fig. 11 | Emissive moieties in synthesized powder. a, Optical image (left) and confocal PL intensity mapping (right) of the 90-min sample observed at the same spatial location. Scale bar on the inset of left image of **a** is 10 μm and scale bar on the right image of **a** is 30 μm. **b,c,** PL intensity mapping (**b**) and PL peak wavelength mapping (**c**) at the location from the magnified inset in **a**. Both scale bars on **b** and **c** are 5 μm. Note the color gradient in **b** is presented in a logarithmic scale. The inset in **b** is the PL spectrum measured on the emission center marked by the white square. All PL measurements are performed under a 405-nm laser excitation source.

Additionally, we conducted a local compositional analysis to investigate the detailed configuration of 3D and 0D phases at the highly luminescent green regions. To this end, the same spatial location on the 90-min sample was probed using optical microscopy and scanning electron microscopy (SEM) (left and right images of Supplementary Fig. 12a, respectively). The green emission is localized on a particle (white-circled region in the optical and SEM images), which was subsequently observed under a higher magnification (Supplementary Fig. 12b). The enlarged optical image in the left image of Supplementary Fig. 12b reveals that the green emitting region (enclosed area indicated by the black dashed line) appears to be surrounded by an external translucent structure. When the atomic ratio is calculated for the green emitting region using energy-dispersive X-ray spectroscopy (EDS) mapping, the Cs:Pb:Br atomic ratio is found to be 4.00:1.22:6.72 (see Supplementary Fig. 12c). Comparing with the stoichiometric ratio of 0D Cs_4PbBr_6 (4:1:6), this atomic ratio refers to the existence of a relatively Pb- and Br-rich phase (or Cs-poor phase), which suggests that the green emitting region contains 3D $CsPbBr_3$. An EDS line scan (indicated by the cyan arrow in Supplementary Fig. 12b) shows that the atomic fraction of Br increased while Cs decreased when approaching the emissive area (see the right image in Supplementary Fig. 12b and Supplementary Fig. 12d). The evolution of the Br/Cs atomic ratio along the distance clearly shows that the green emitting region has a higher Br/Cs ratio, suggesting that the green emitting part contains higher portion of 3D $CsPbBr_3$ than the peripheral area (outside of the black dashed circle in Supplementary Fig. 12b). Note that the EDS line scan showed a relatively low signal-to-noise ratio for Pb (which is to be discussed on Supplementary Fig. 13). Hence, all of these results in combination support the presence of 3D $CsPbBr_3$ nanocrystals encapsulated within 0D Cs_4PbBr_6 matrix as the source of green emission in the 0D samples.

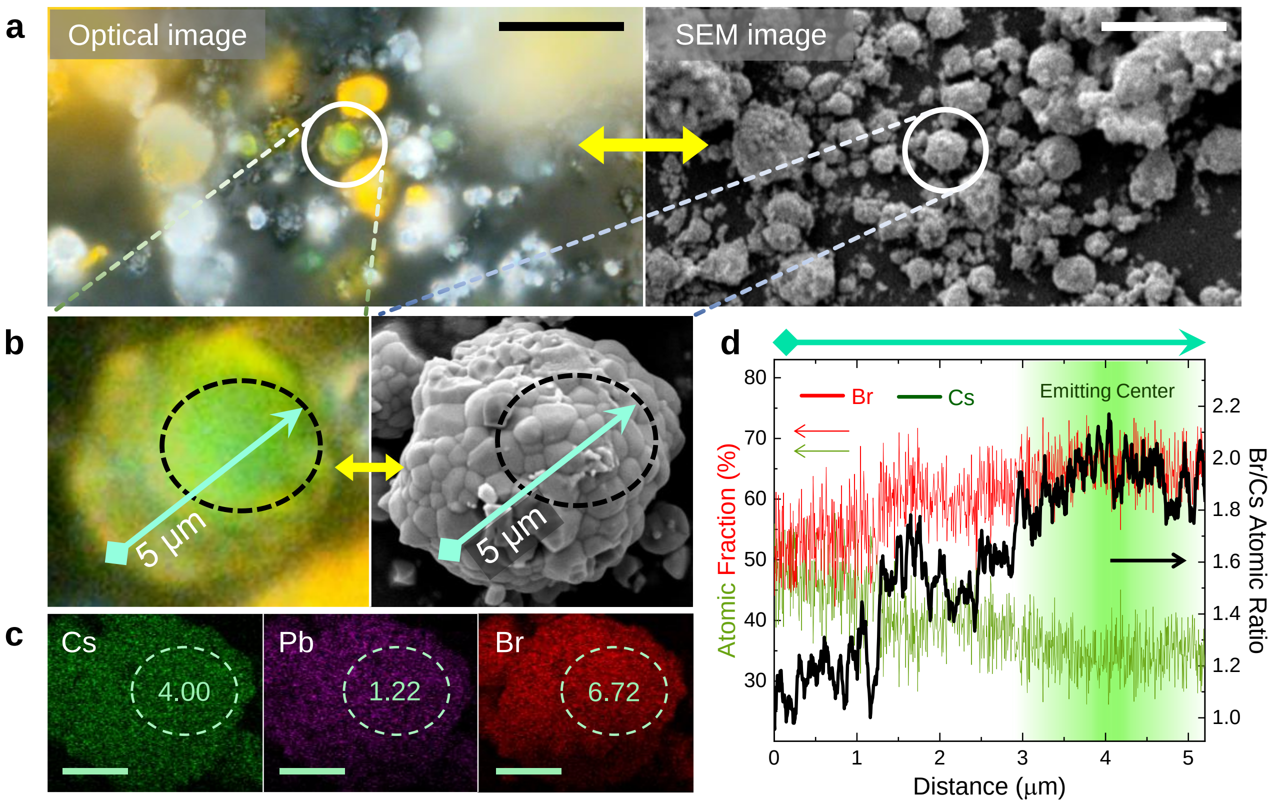

Supplementary Fig. 12 | Microscopic examination of bright green regions in the synthesized powder. **a,** Optical (left) and SEM (right) images observed at the same spatial location. Scale bar, 15 μm. **b,c,** Magnified optical image (**b**; left), SEM image (**b**; right), and EDS elemental mapping (**c**) of Cs, Pb, and Br in the white-circled regions in **a**. Scale bar, 2 μm. All the dashed black circles in **b** and dashed cyan circles in **c** are marked at the same spatial location as an indication of the green emissive region. Numbers written inside the dashed cyan circles in **c** are the atomic ratio of elements calculated through partial EDS mapping in the dashed black circle from the SEM image in **b**. **d,** EDS line scan of Cs (green), Br (red), and Br/Cs atomic ratio (black) over the cyan arrow in the SEM image in **b**.

As shown in Supplementary Fig. 13, detection of Pb M series in energy-dispersive X-ray spectroscopy (EDS) line scan was highly unstable compared to Cs L series and Br L series, where diverse spots were detected to have zero fraction of Pb. Moreover, since the quantitative EDS analysis is more reliable for elements with higher proportions[19], we analyzed the ratio of Br to Cs in Supplementary Fig. 12d when contrasting the atomic composition of the emitting region to the peripheral region. Line scan procedure had 5.6 nm of spatial resolution.

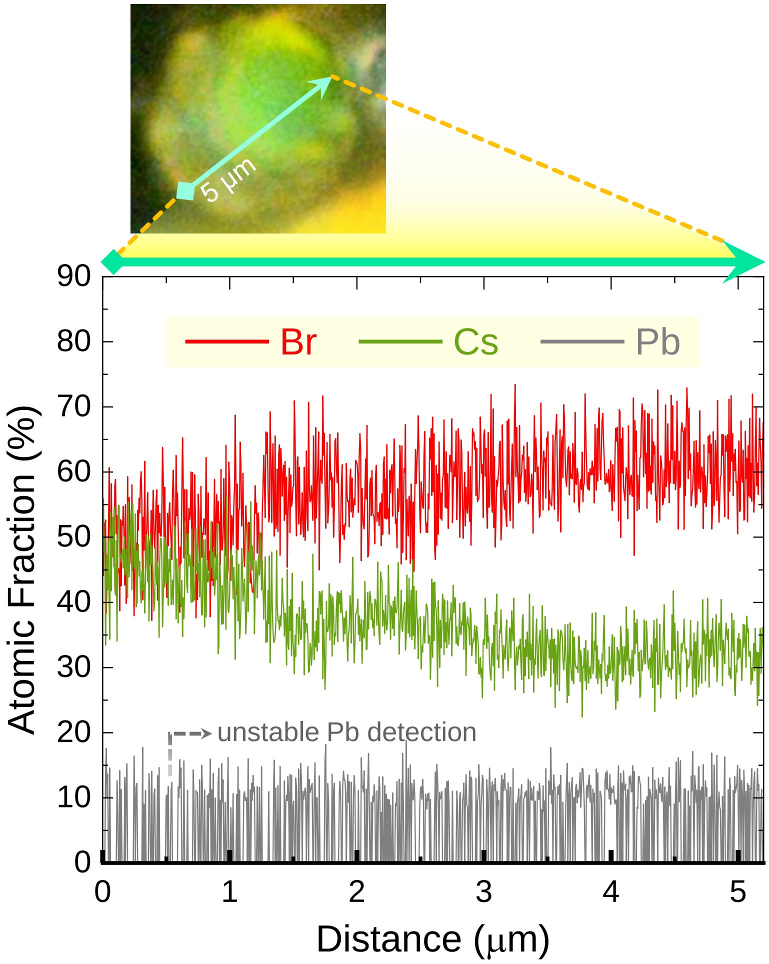

Supplementary Fig. 13 | EDS line scan. Atomic fraction of Br, Cs, and Pb is examined over the line marked as cyan arrow. Optical image on the top is an identical image from the left image of Supplementary Fig. 12b.

2.3 Exciton binding energy of the 2880-min sample

We characterized the PL peak (at 507 min, see Supplementary Fig. 6) of the 2880-min sample by measuring the temperature-dependent confocal PL of the white Cs_4PbBr_6 grains, as shown in Supplementary Fig. 14. The exciton binding energy of the PL peak was found to be 225.7 ± 18.6 meV from the Arrhenius fitting. The large 225.7 meV value suggests a tightly bound Frenkel excitonic property and it is known as the value for 0D Cs_4PbBr_6 (refs. [5,8,20,21]). Along with the results from Fig. 2f of the main text where a similar process was conducted on 90-min sample, we can assure that high-intensity PL in the intermediate synthesis steps can be attributed to the presence of 3D $CsPbBr_3$.

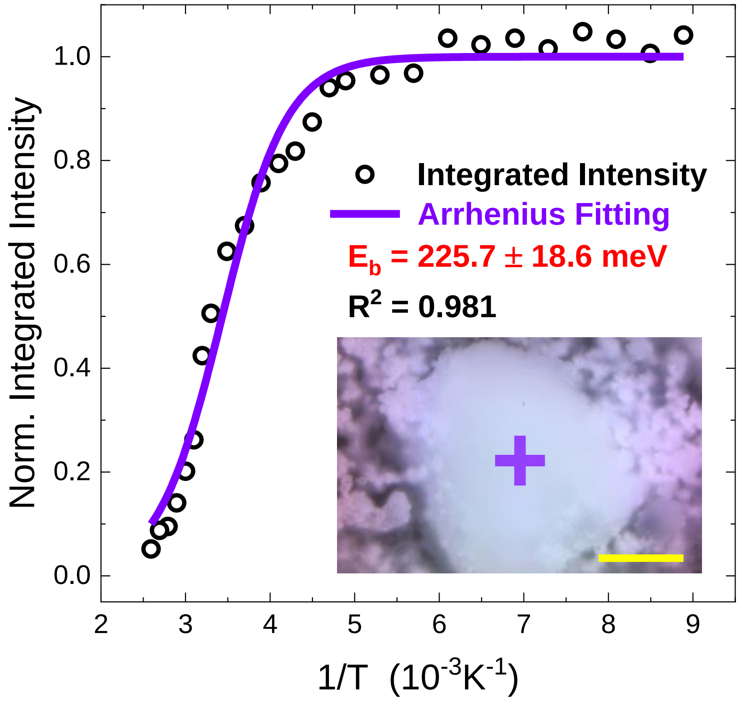

Supplementary Fig. 14 | Exciton binding energy of the synthesis completed sample. Temperature-dependent integrated PL intensity of the 507-nm PL peak in the 2880-min sample under a 405-nm laser excitation source. The inset shows an optical image of the target scene with laser collimation marked in purple cross. Scale bar, 50 μm.

2.4 SEM observation and EDS elemental mapping

Supplementary Figure 15 shows scanning electron microscopy (SEM) images of the samples from different milling times under identical magnification. We can observe the gradual change from coarse particles to fine particles with successive increase in milling time.

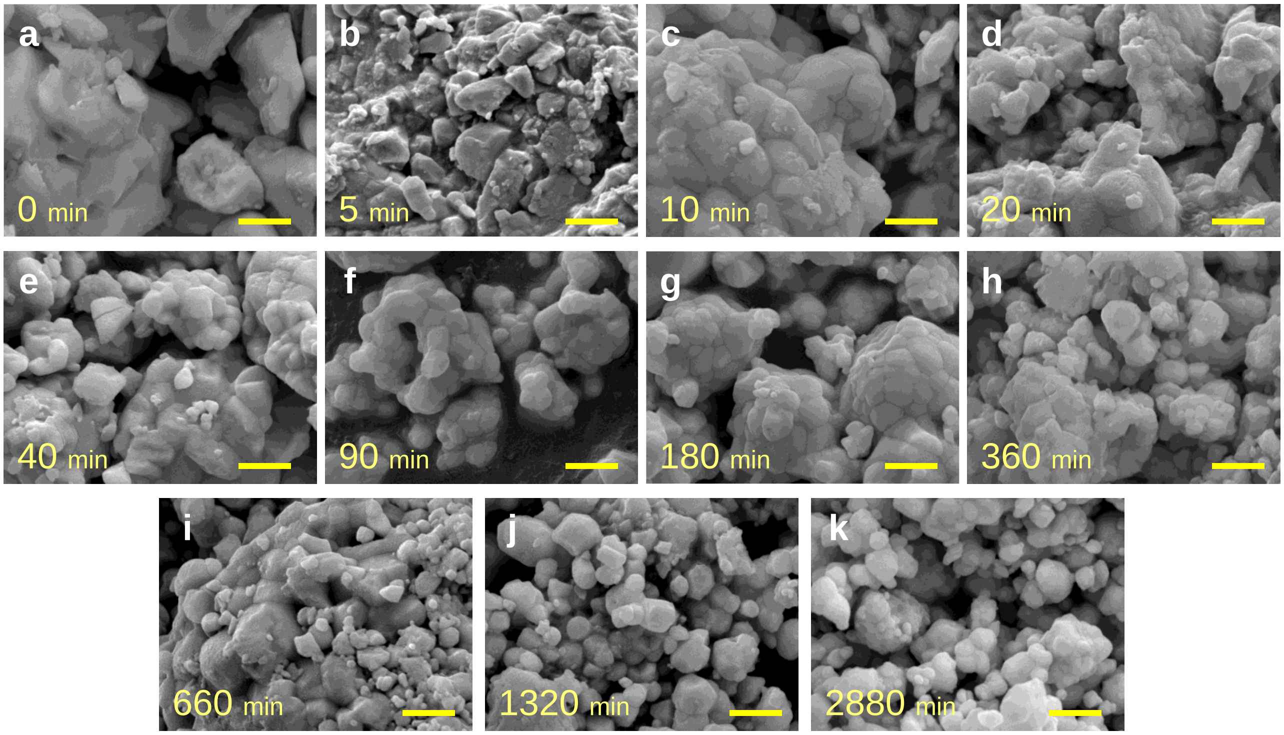

Supplementary Fig. 15 | SEM observation of powder samples. a–k, SEM images of powder samples with different milling times from 0 min to 2880 min. All scale bars, 1 μm.

Supplementary Figure 16 shows SEM images along with EDS mapping images of the pure 3D $CsPbBr_3$ and 2D $CsPb_2Br_5$ which are mechanochemically synthesized through our rotating mill-type ball mill equipment. Recipe for the preparation of each sample follows the first subsection in the Methods section. According to EDS mapping results, Cs:Pb:Br atomic ratio of the synthesized 3D $CsPbBr_3$ is 1.00:1.04:2.91 and 2D $CsPb_2Br_5$ is 1.00:2.04:4.76. Comparing with the stoichiometric ratio of 3D $CsPbBr_3$ (1:1:3) and 2D $CsPb_2Br_5$ (1:2:5), it proves the high purity of the samples.

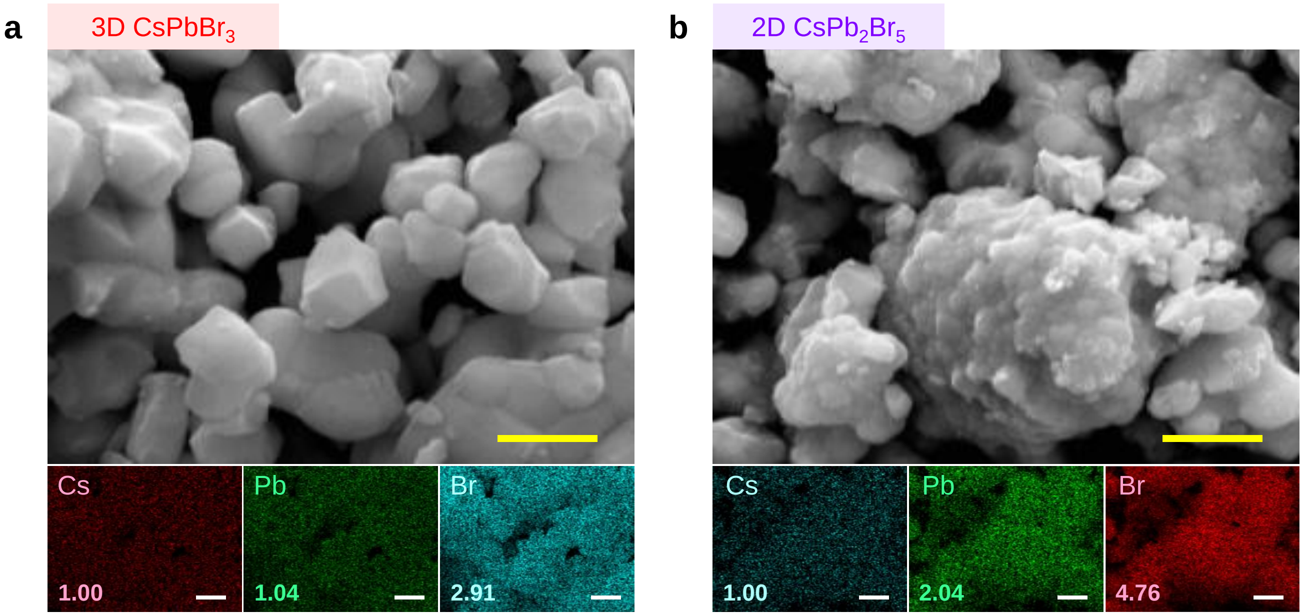

Supplementary Fig. 16 | Atomic mapping of powder samples. a,b, SEM images and the corresponding EDS mapping images of fully synthesized 3D $CsPbBr_3$ (**a**) and 2D $CsPb_2Br_5$ (**b**) and the numbers shown in bottom-left part of each EDS mapping image are the atomic ratio of each atom. All scale bars (both yellow and white), 1 µm.

Supplementary Figure 17 shows SEM images and corresponding EDS mapping images of powder samples with different milling times in the synthesis process of 0D Cs_4PbBr_6. We can distinguish the unreacted precursors in 5-min and 90-min samples (Supplementary Figs. 17a and 17b) through observing the colors of each portion which is marked as dotted cyan circles for CsBr and dotted yellow circles for $PbBr_2$. For example, when observing the entity marked as dotted yellow circles in Supplementary Fig. 17a, colors corresponding to Pb and Br (purple and red, respectively) are revealed whereas Cs mapping image shows only black color. This means that the entity only contains Pb and Br atoms, thereby concluding that this part is $PbBr_2$. On the other hand, starting from 180-min sample (Supplementary Fig. 17c), atoms of Cs, Pb, and Br are detected uniformly over the entire area under micrometer-scale. In the 2880-min sample (Supplementary Fig. 17d) which is the synthesis-completed sample, size average of the synthesized grains is observed to be ~533 nm and EDS mapping results present that the Cs:Pb:Br atomic ratio of synthesized portions is 4.00:1.05:6.72. Supplementary Figure 17e is the EDS energy spectrum of the powder from 2880-min sample.

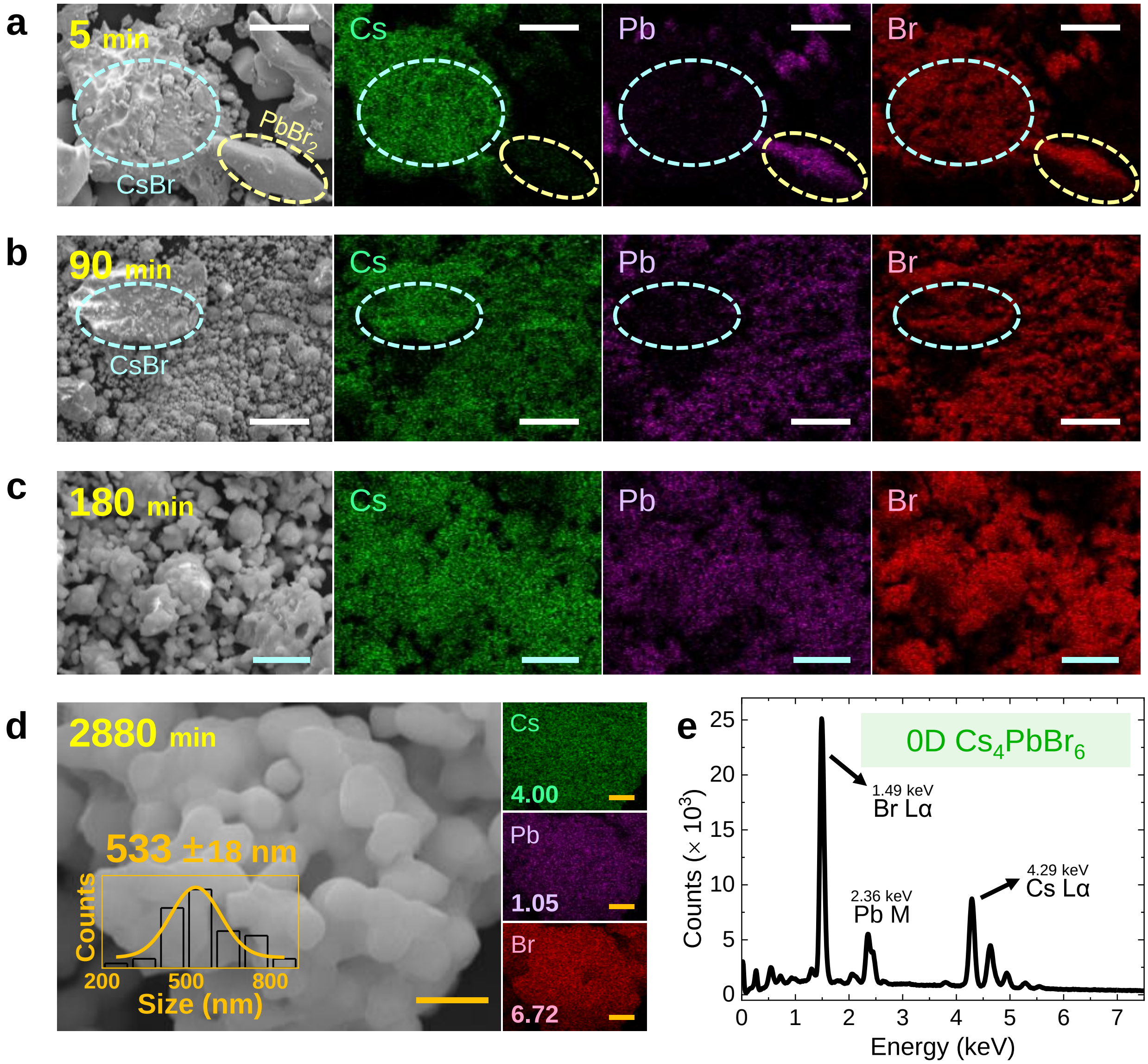

Supplementary Fig. 17 | SEM and EDS mapping images of samples with different milling times in the synthesis process of 0D Cs_4PbBr_6. a–d, SEM image and corresponding EDS mapping images of different atoms from 5-min (**a**), 90-min (**b**), 180-min (**c**), and 2880-min (**d**) samples. Precursors of CsBr and $PbBr_2$ are marked as dotted cyan circles and dotted yellow circles, respectively. For all EDS mapping images, the black color indicates the absence of detection for the corresponding atom. **e,** EDS energy spectrum from 2880-min sample. White scale bars in **a** and **b**, 25 µm. Cyan scale bars in **c**, 5 µm, Orange scale bars in **d**, 1 µm.

Supplementary Note 3: Time evolution of structural properties during MCS process

3.1 Time evolution of PXRD and NMR spectra

Supplementary Figure 18 shows the time evolution of PXRD patterns in the full spectrum range of 10 to 50 degrees. Figure 3a from the main text is an enlarged view from 10 to 32 degrees from this graph.

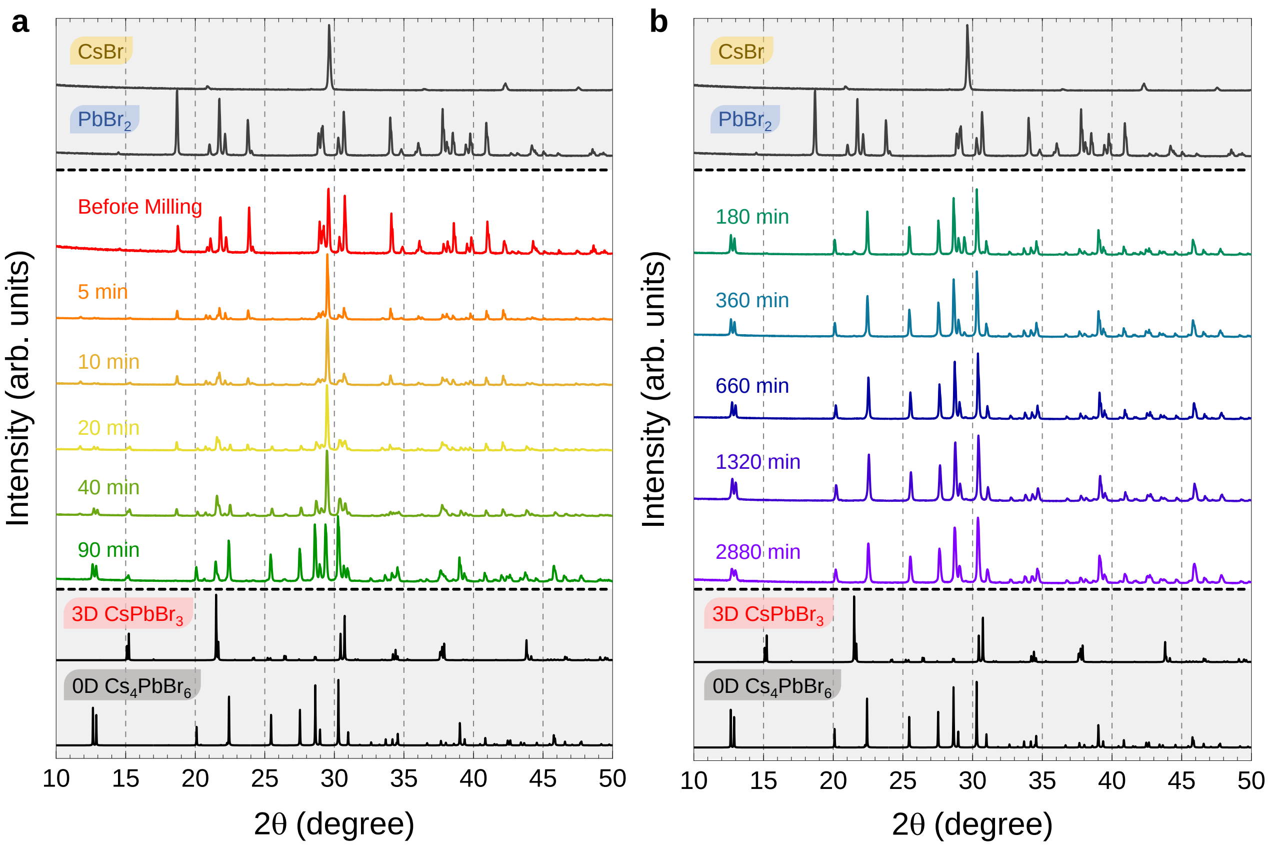

Supplementary Fig. 18 | Time evolution of PXRD patterns. a,b, PXRD data until 90 min of milling time (i.e., Stage 1 of the synthesis process; **a**) and afterwards (i.e., Stage 2 of the synthesis process; **b**). PXRD patterns of two precursors (CsBr and $PbBr_2$) are measured values, and 3D $CsPbBr_3$ and 0D Cs_4PbBr_6 are reference patterns from COD ID 4510745 (ref. [4]) and 4002857 (ref. [5]), respectively.

On the same note, Supplementary Fig. 19 shows the time evolution of solid-state nuclear magnetic resonance (ssNMR) patterns in the full spectrum range of −300 to 700 ppm. Figure 3b from the main text is an enlarged view from −100 to 400 ppm.

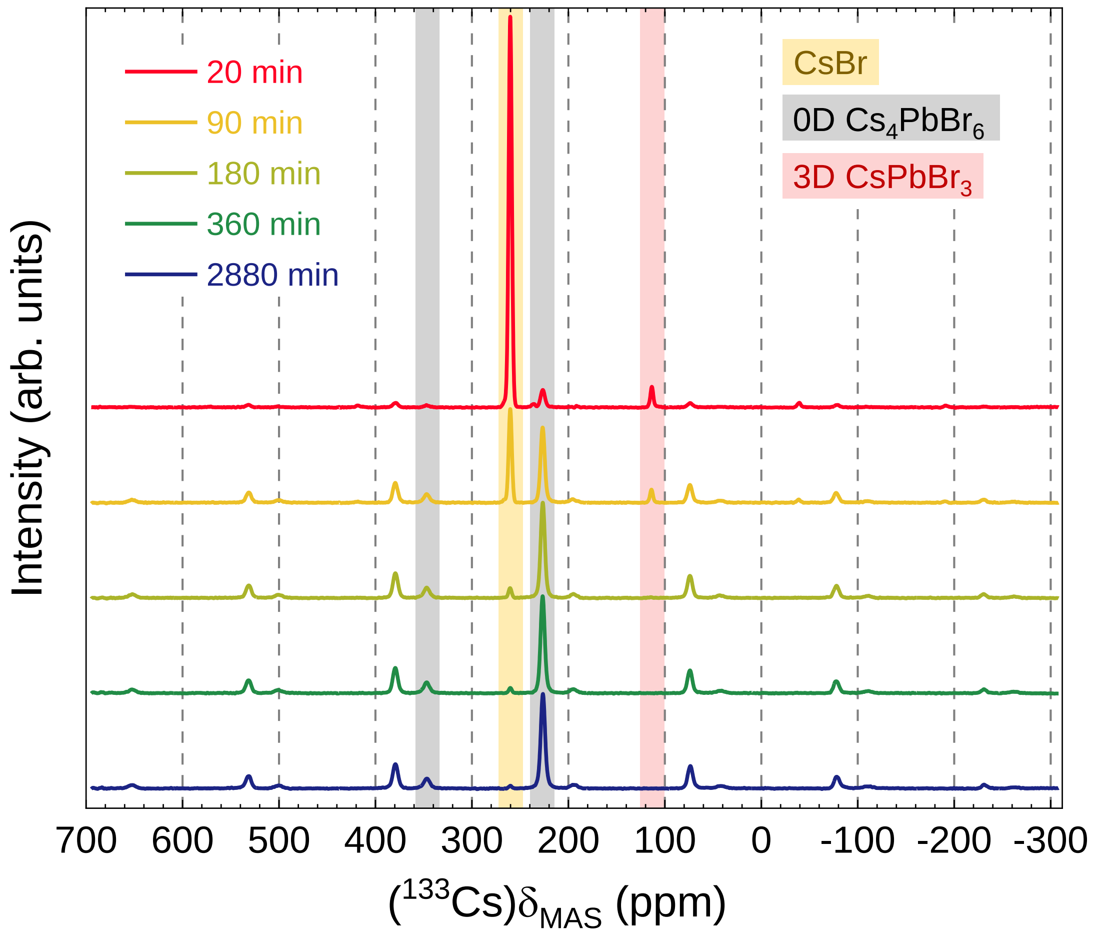

Supplementary Fig. 19 | Time evolution of ssNMR patterns. ssNMR data of samples from five different milling times (20, 90, 180, 360, and 2880 min). CsBr, 0D Cs_4PbBr_6, and 3D $CsPbBr_3$ peaks are marked with semi-transparent yellow, black, and red strips, respectively. The remaining peaks correspond to spinning side bands.

Supplementary Figure 20 shows the evolution of figure-of-merit (FOM) values of 3D $CsPbBr_3$ and 0D Cs_4PbBr_6 calculated by PDXL software, Rigaku. FOM value is an indication of how much the PXRD pattern from Supplementary Fig. 18 of each milling step matches the patterns of 3D $CsPbBr_3$ or 0D Cs_4PbBr_6. It is calculated by the following formula[22]: $F_N = (1/\Delta_{2\theta})(N_{obs}/N_{poss})$, where F_N is the FOM, $\Delta_{2\theta}$ is the average error in 2θ, N_{obs} is the number of lines observed, and N_{poss} is the number of lines possible. Overall, the magnitude of FOM is inversely proportional to the matching degree: lower FOM value corresponds to a better match of each reference and higher FOM value corresponds to a poor match of each reference. When observing the time evolution in FOM values of 3D $CsPbBr_3$ (red diamond symbol in Supplementary Fig. 20), it can be seen that the content of 3D $CsPbBr_3$ gradually increases (since the FOM value decreases) until 90 min of milling time and then decreases thereafter (since the FOM value increases). Moreover, when observing the time evolution in FOM values of 0D Cs_4PbBr_6 (black triangle symbol in Supplementary Fig. 20), it can be seen that the content of 0D Cs_4PbBr_6 gradually increases (since the FOM value decreases) towards the completion of synthesis. These results corroborate the trend observed in Fig. 4b from the main text.

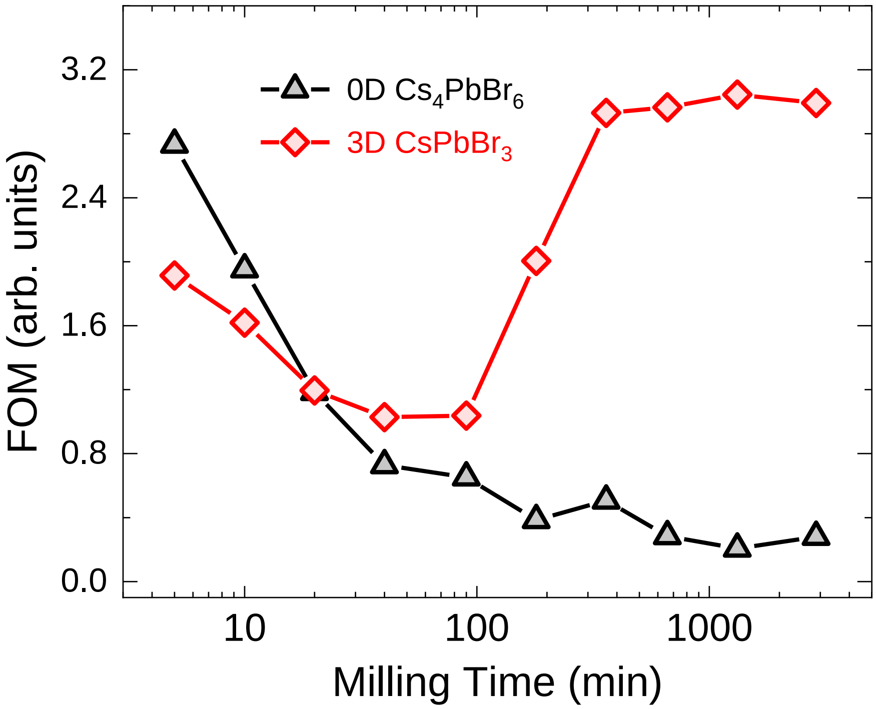

Supplementary Fig. 20 | Compositional evolution in FOM. Evolution of FOM values of (red diamonds) 3D $CsPbBr_3$ and (black triangles) 0D Cs_4PbBr_6.

Supplementary Figure 21 shows the molar fractions of precursors and synthesized Cs–Pb–Br perovskites for each sample calculated through Rietveld refinement process. The same graph shown in weight fraction was presented in Fig. 4b of the main text.

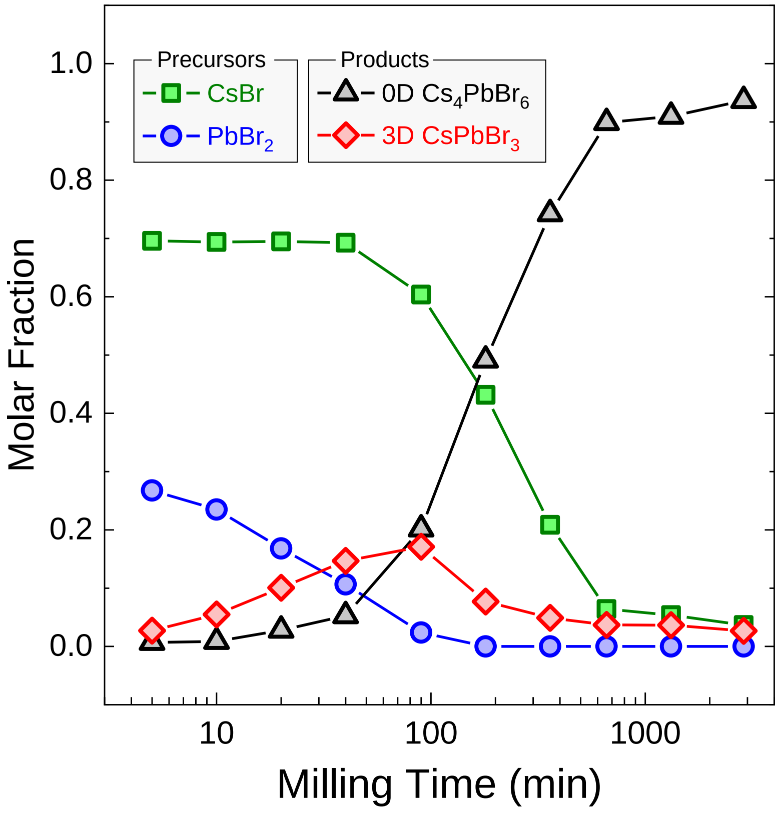

Supplementary Fig. 21 | Compositional evolution in molar fraction. Molar fractions of compositions in each sample with milling time from 5 min to 2880 min.

Supplementary Figure 22 shows the time evolution of weight fraction and molar fraction when Stage 1 (until 90 min of milling time) and Stage 2 (from 180 min to 2880 min of milling time) are synthesized separately, i.e., samples of Stage 2 are not the time-extensions of Stage 1 but are newly milled samples. These results demonstrate the reproducibility of characteristics which were discussed in Fig. 4b of the main text: the formation rate of 3D $CsPbBr_3$ is faster than 0D Cs_4PbBr_6 in Stage 1 and the as-synthesized $CsPbBr_3$ react with the remaining CsBr to produce Cs_4PbBr_6 in Stage 2.

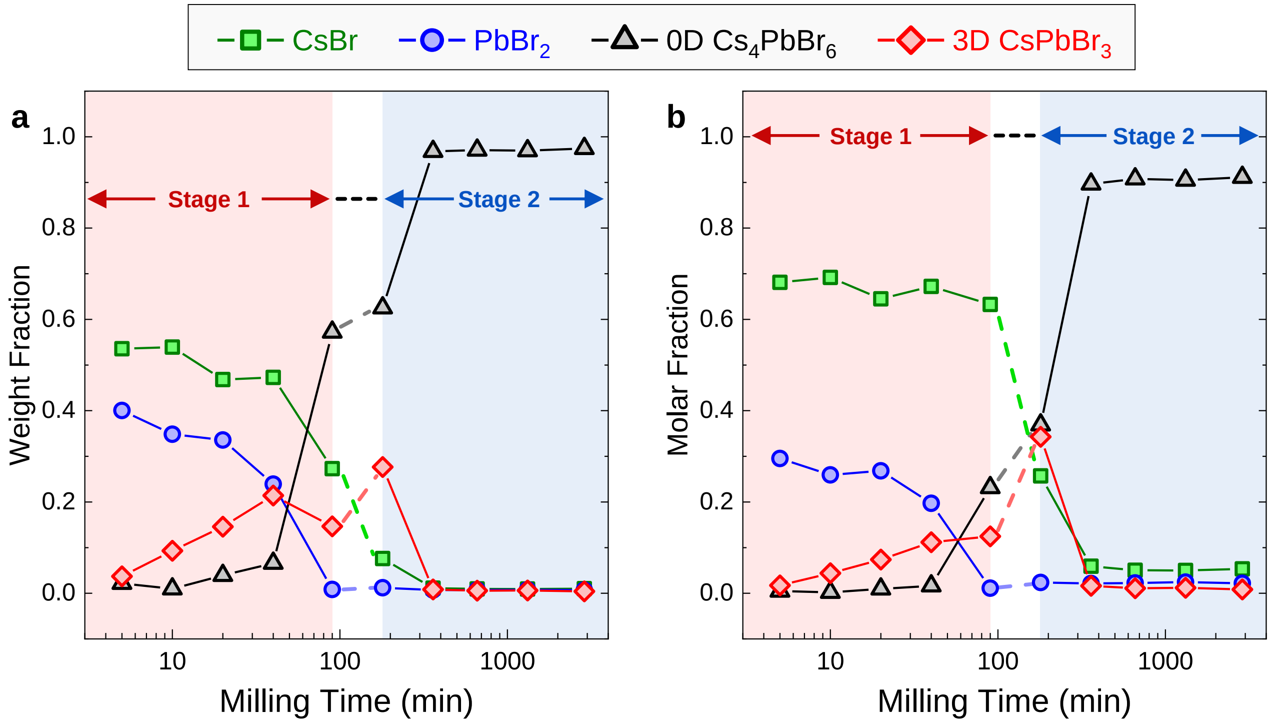

Supplementary Fig. 22 | Reproducibility in compositional evolution. a,b, Time evolution of weight fraction (**a**) and molar fraction (**b**) when samples of Stage 1 and Stage 2 are synthesized separately.

3.2 Insights on defect-related emission via Raman spectroscopy

Since the Raman active vibration modes of 3D $CsPbBr_3$ and 0D Cs_4PbBr_6 are known to differ due to their distinct $[PbBr_6]^{4-}$ octahedra interconnectivity[23,24], Raman spectroscopy allows to detect trace amount of 3D $CsPbBr_3$ particles in the nominal 0D Cs_4PbBr_6 powder with higher sensitivity than the PXRD method. Supplementary Figure 23 shows the Raman spectroscopy measurements of 660-min and 2880-min samples under liquid nitrogen temperature (77.5 K). Each measurement is focused on white grains where Cs_4PbBr_6 is expected to be the sole product. Notable discrepancy between the two samples arises from the Raman band around 310 cm^{-1}. This peak arises from the second-order phonon scattering mode of the octahedron in 3D $CsPbBr_3$ perovskite structure[25,26], which provides direct evidence of $CsPbBr_3$ phase[23] in the 660-min sample. While PXRD pattern of the 660-min sample showed no indication of $CsPbBr_3$-related peaks (Fig. 3 from the main text and Supplementary Fig. 18), Raman spectroscopy confirms the presence of trace-level-3D $CsPbBr_3$ within the nominally 0D Cs_4PbBr_6 phase which is under the detection limit of X-ray diffractometer. On the other hand, the Raman spectrum of the 2880-min sample does not show the presence of the 310 cm^{-1} peak, indicating the absence (within the Raman detection limit) of 3D phase in the synthesis-completed sample, consistent with the emission measurements. Raman peaks on low frequency region (< 200 cm^{-1}) of both samples are attributed to Pb–Br rocking modes of 0D Cs_4PbBr_6 perovskite structure[14,27].

Regarding the alternative origin of green emission in 0D Cs_4PbBr_6, the polybromide model is drawing attention as a possible emission source based on modern first principles theory of defects and excited state calculations[28]. This model attributes a portion of green emission to the formation of tribromide (Br_3^-) species which results from Br-on-Cs anti-site defects. While this tribromide species is expected to have a Raman active vibrational mode at 168 cm^{-1} (v_3 mode of Br_3^-), this band is not clearly observed in our measurements in addition to refs. [17,23,24] that only seem to show a mere trace of shoulder

peak at around 160 cm^{-1} (see the gray dashed arrow in Supplementary Fig. 23). While other evidences as presented in the main text suggest that the green emission in our sample could primarily be attributed to 3D $CsPbBr_3$ phase, we note that our results do not completely exclude the polybromide defect scenario as the possible explanation for the source of green emission: the lack of sufficient resolution in the Raman spectrum arising from the overlap of 168 cm^{-1} band with the Pb–Br stretching mode (151 cm^{-1}) of 0D Cs_4PbBr_6 perovskite structure[14] can be accounted for the obscure signal. A more detailed analysis will be required to experimentally reveal the existence of such type of defect.

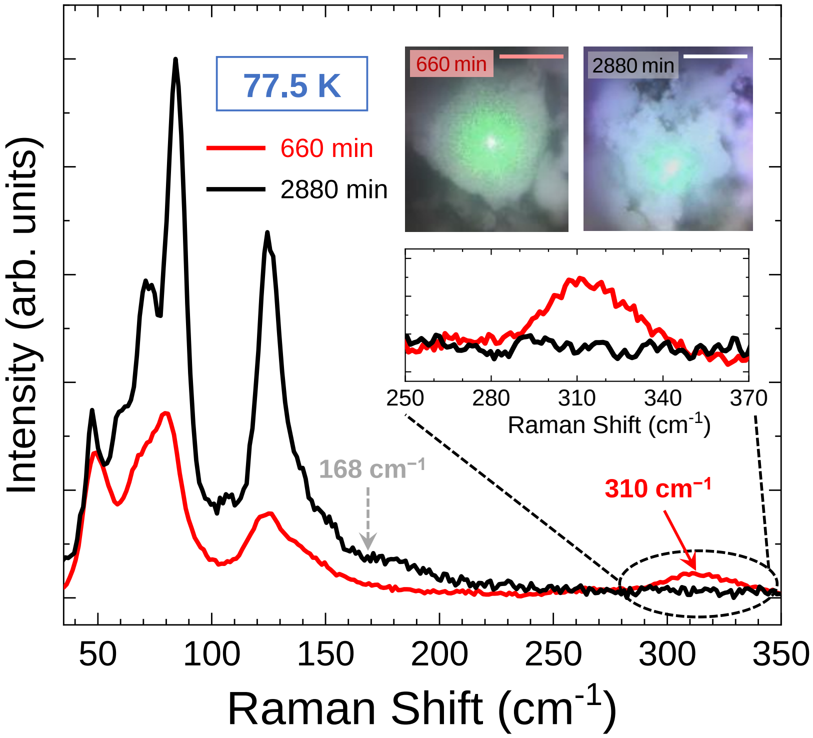

Supplementary Fig. 23 | Raman spectrum of 660-min and 2880-min samples measured at 77.5 K and excited with 532 nm laser. Two insets on upper-right show optical images of each sample and green spots are the laser points. The 310 cm^{-1} peak is an indication of 3D $CsPbBr_3$ presence. Both scale bars on insets, 50 μm.

3.3 Examining the 2D $CsPb_2Br_5$-related emission

Following the reaction formula $CsBr + 2PbBr_2 \rightarrow CsPb_2Br_5$, we examined the time evolutional properties in the synthesis process of 2D $CsPb_2Br_5$ (i.e., one of the stable phases of Cs–Pb–Br ternary system[29–31]) in order to exclude the effect of $CsPb_2Br_5$ on the observed green PL. Supplementary Figure 24a shows the image of powder samples over 5 different milling times (10, 90, 660, 2880, and 5760 min). 5760-min sample is the pure 2D $CsPb_2Br_5$ powder which will be verified later. When observed under UV lamp of 365-nm wavelength, there was almost no detection of visible light under the naked eye. This is in direct contrast to Fig. 2a from the main text where a clear gradient of PL intensity change was detected. In order to emphasize the difference in intensity when viewed through a UV lamp, Supplementary Fig. 24b is a side-by-side photograph with 180-min sample from Fig. 2a (i.e., the brightest sample in the process of synthesizing 0D Cs_4PbBr_6). Supplementary Figure 24c shows the PL and PLE measurements through spectrofluorometer and it shows that the PL with peak wavelength of 523 nm is detected for the first three steps (i.e., 10, 90, and 660-min samples). The reason that green PL (which was not visible to the naked eye) is detected here is that the intensity of excitation source in the spectrofluorometer is higher and the detector is also more sensitive than the human eye. Nevertheless, it is clear that the overall PL intensity in the synthesis process of 2D $CsPb_2Br_5$ is not as strong as when synthesizing 0D Cs_4PbBr_6. Absorption spectra shown in Supplementary Fig. 24d presents a clear onset near 550 nm for the first three samples for which we can assume that they contain a portion of 3D $CsPbBr_3$. Structural properties observed through PXRD (Supplementary Fig. 24e) and weight fractions derived through Rietveld refinement process (Supplementary Fig. 24f) further prove that the first three samples contain 3D $CsPbBr_3$. All these results imply that the detected green PL here is also due to 3D $CsPbBr_3$, and 2D $CsPb_2Br_5$ itself cannot act as an efficient emitting source. Therefore, we can assert that the trace amount of 2D $CsPb_2Br_5$ detected ssNMR analysis in Fig. 4a from the main text has negligible effect on the overall green PL.

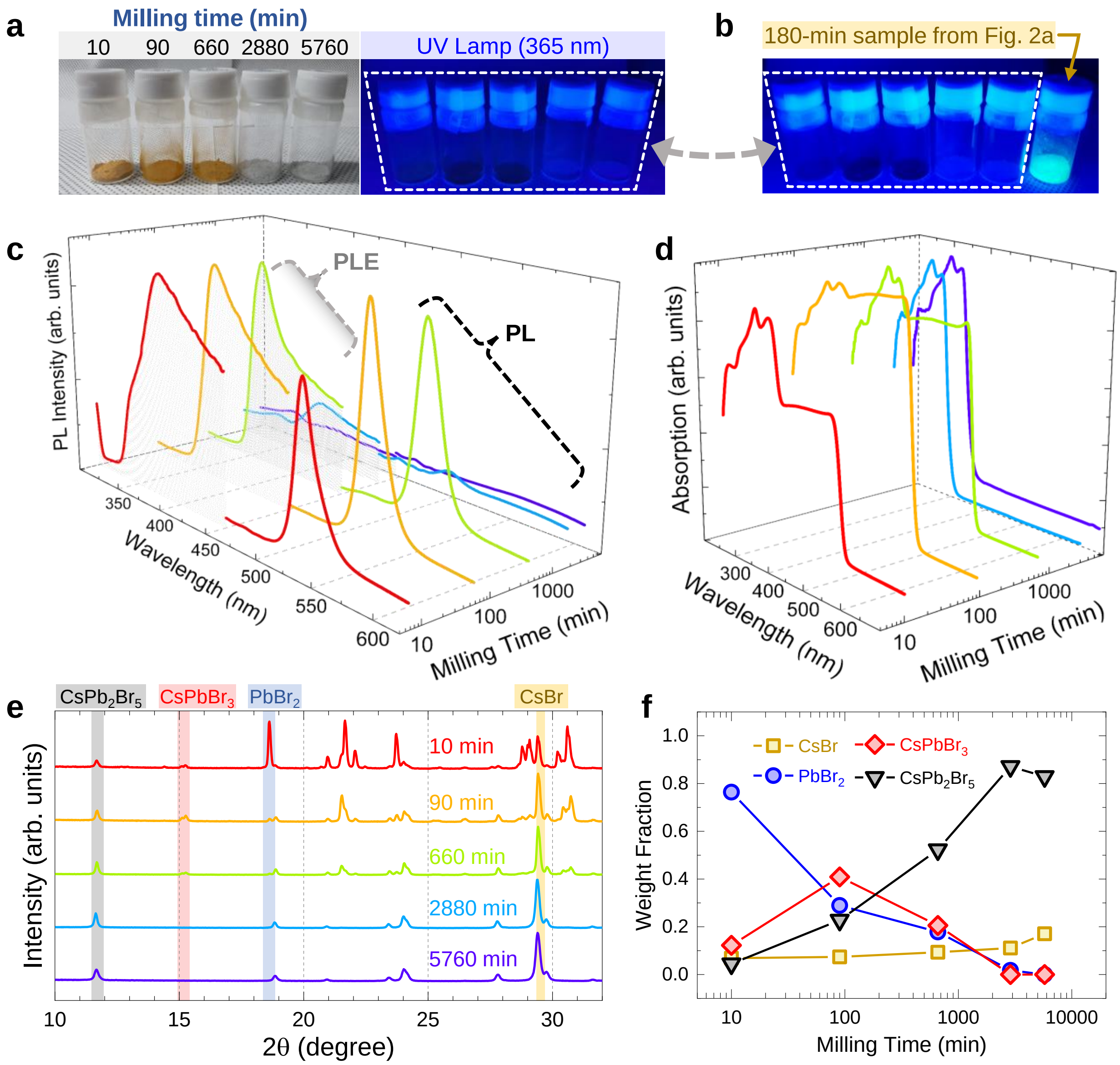

Supplementary Fig. 24 | Time evolutional properties in the synthesis process of 2D $CsPb_2Br_5$. a, Images of powder samples under ordinary fluorescent (top) and UV (bottom) lamps. **b,** Image of powder samples along with the 180-min sample from Fig. 2a of the main text under UV lamp. **c,d,** Time evolution of PL (with 365-nm excitation; **c**) and PLE (at 525-nm emission; **c**), and absorption spectra (**d**). **e,** Time evolution of PXRD patterns. Representative peaks of $CsPb_2Br_5$, $CsPbBr_3$, $PbBr_2$, and CsBr are marked by semi-transparent black, red, blue, and yellow strips, respectively. **f,** Weight fractions of compositions in each sample calculated through Rietveld refinement process.

Supplementary Note 4: Designing a synthesis route for highly emissive perovskite

4.1 Phase transformation between 0D Cs_4PbBr_6 and 3D $CsPbBr_3$ through MCS

As MCS method allows us to use different solid-state precursors, here we aim to show an interconversion between the two perovskite phases (0D Cs_4PbBr_6 and 3D $CsPbBr_3$) through ball-milling with additional reactants ($PbBr_2$, CsBr). Supplementary Figure 25 shows the feasibility of phase transformation between 3D $CsPbBr_3$ and 0D Cs_4PbBr_6 when added with appropriate precursor of desired stoichiometry (refer to reaction formulas, $CsPbBr_3 + 3CsBr \rightarrow Cs_4PbBr_6$ and $Cs_4PbBr_6 + 3PbBr_2 \rightarrow 4CsPbBr_3$). Phase transformation from 3D $CsPbBr_3$ to 0D Cs_4PbBr_6 is presented in Supplementary Figs. 25a–c and phase transformation in the opposite direction is presented in Supplementary Figs. 25d–f. Images in Supplementary Figs. 25b and 25d show the powder along with stainless-steel grinding balls before the initiation of synthesis. Color change of the powder samples between orange color of 3D $CsPbBr_3$ and white color of 0D Cs_4PbBr_6 is clearly visible. All precursors (CsBr and $PbBr_2$) possess white color. Agreements between the observed and simulated PXRD patterns as shown in Supplementary Figs. 25a, 25c, 25d, and 25f prove the precision as well as the versatility of realizing phase transformation through MCS process. Hence, this result demonstrates the possibility of realizing endotaxial growth of 0D phases from the 3D phase powder (and vice versa) as shown in the main text (refer to Figs. 4a and 4c of the main text).

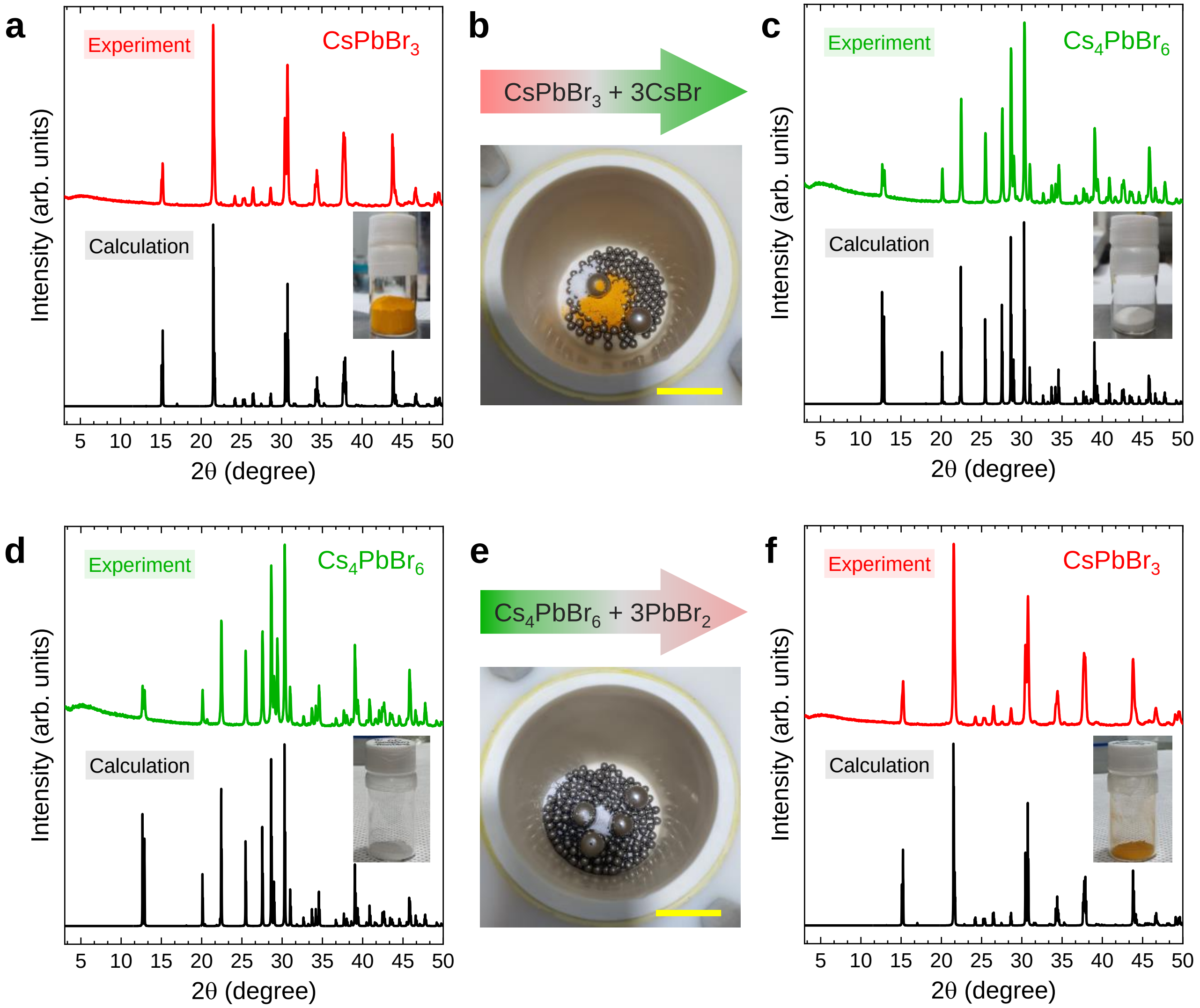

Supplementary Fig. 25 | Phase transformation realized through MCS. a–c, Phase transformation from $CsPbBr_3$ to Cs_4PbBr_6 with the addition of CsBr. **d–f,** Phase transformation from Cs_4PbBr_6 to $CsPbBr_3$ with the addition of $PbBr_2$. Simulated PXRD patterns of $CsPbBr_3$ and Cs_4PbBr_6 are from COD ID 4510745 (ref. [4]) and 4002857 (ref. [5]), respectively. Scale bars on **b** and **e** are 3 cm.

4.2 Optimizing the composition ratio between 0D Cs_4PbBr_6 and 3D $CsPbBr_3$

In order to find the optimal ratio between 0D Cs_4PbBr_6 and 3D $CsPbBr_3$ in the final product to synthesize 0D/3D heterostructure with the highest PL emissive properties, we varied the stoichiometric number ($\boldsymbol{x}$) of CsBr as shown in the reaction formula in Supplementary Fig. 26a. We aimed to produce 1.0 to 15.0 wt% of 3D $CsPbBr_3$ with respect to the total mass of the final product and the $\boldsymbol{x}$ value for actualizing each weight fraction is calculated and presented in Supplementary Fig. 26b.

a $$CsPbBr_3 + x\,CsBr \rightarrow (x/3)\,Cs_4PbBr_6 + (1 - x/3)\,CsPbBr_3$$

b

Weight fraction of $CsPbBr_3$	x
**1.0 wt%**	*2.94*
**3.0 wt%**	*2.82*
**5.0 wt%**	*2.70*
**7.0 wt%**	*2.59*
**9.0 wt%**	*2.48*
**11.0 wt%**	*2.38*
**13.0 wt%**	*2.28*
**15.0 wt%**	*2.19*

Supplementary Fig. 26 | Synthesizing powder samples of 0D/3D heterostructure with controlled weight fraction. a, Reaction formula when 3D $CsPbBr_3$ is synthesized with an arbitrary stoichiometry (x) of CsBr. **b,** Calculated x values in order to realize the desired weight fractions.

Supplementary Figure 27 shows PXRD patterns of the synthesized samples with different weight fractions of 3D $CsPbBr_3$ as designed in Supplementary Fig. 26b.

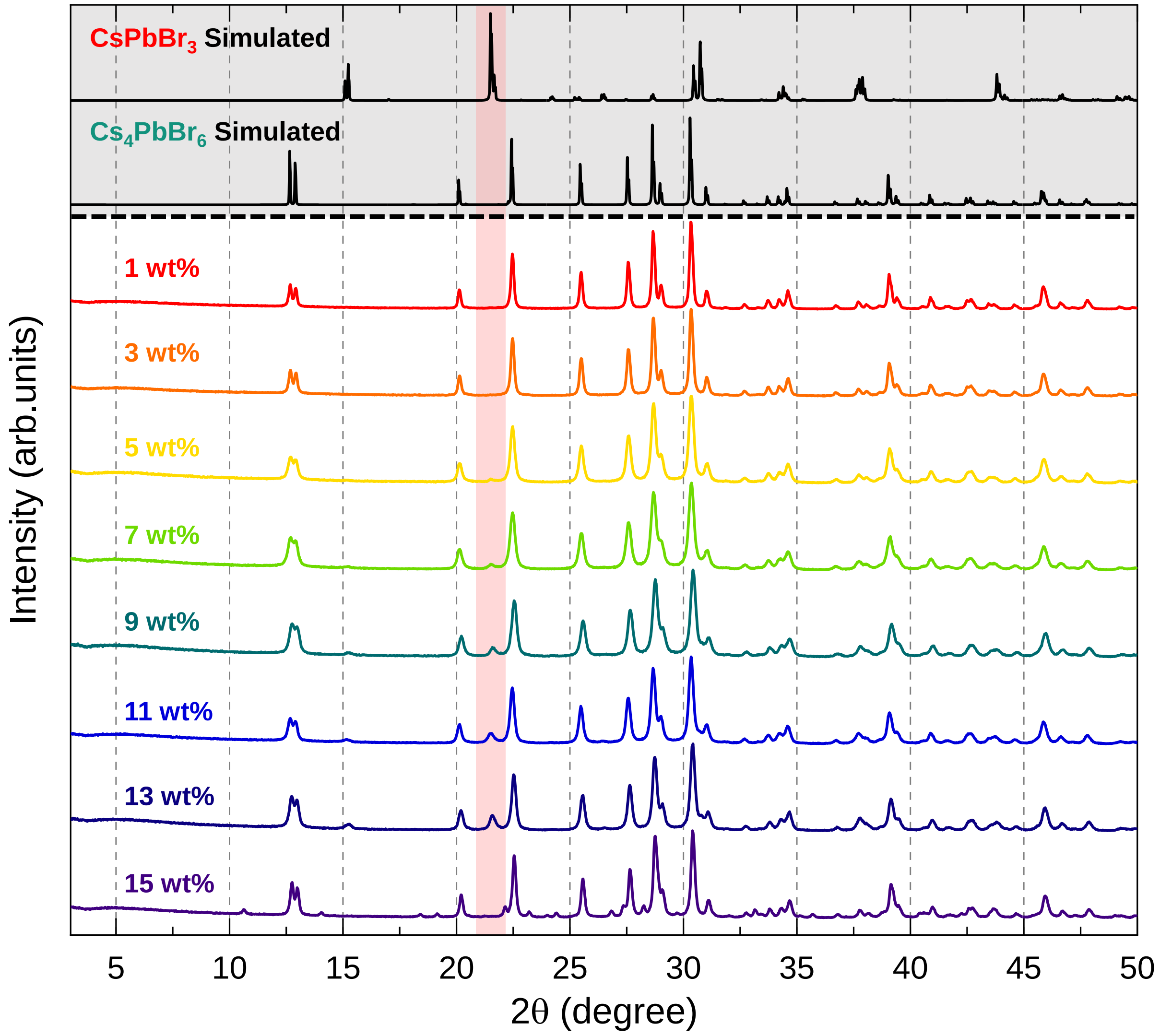

Supplementary Fig. 27 | PXRD patterns of samples with varying compositional ratio of 3D. PXRD patterns of synthesized samples with weight fraction of 3D $CsPbBr_3$ ranging from 1.0 to 15.0 with an interval of 2.0. Representative peak of 3D $CsPbBr_3$ is marked as semi-transparent red strip. Simulated results of $CsPbBr_3$ and Cs_4PbBr_6 are from COD ID 4510745 (ref. [4]) and 4002857 (ref. [5]), respectively.

Supplementary Figure 28a shows PLE spectra of powder samples with different 3D $CsPbBr_3$ weight fraction ranging from 1.0 to 15.0 with an interval of 2.0. PLE spectra are monitored at emission wavelength of 523 nm. Similar to Supplementary Fig. 7a, two peaks of 294 nm and 365 nm are observed and the space in between shows the "hole-burning" region. Peak intensities show high values for 7, 9, 11, and 13 wt% samples compared to the rest of samples. PL spectra under excitation wavelength of 365 nm are presented in Supplementary Fig. 28b. All spectra show peak position wavelength of 523 nm. Similar to the PLE spectra, peak intensities in PL spectra also show high values for 7, 9, 11, and 13 wt% samples compared to the rest of samples. Supplementary Figure 28c shows PLQE of the samples and reveals that among four samples with high values of peak intensity (i.e., 7, 9, 11, and 13 wt% samples), 7 wt% sample possesses the highest value. It is worth mentioning that the PLQE measurements here are also conducted via packing the powder directly into a quartz cell with a width of 2 mm, similar to the measurements shown in Fig. 2b of the main text. Overall, 0D/3D hetero-structured powder sample with 3D $CsPbBr_3$'s weight fraction of 7 wt% possesses the optimal emissive properties. Therefore, in order to compare the different synthesis routes named as Cases 1, 2, and 3 in Fig. 5a from the main text, all final products were aimed to contain 7 wt% of 3D $CsPbBr_3$.

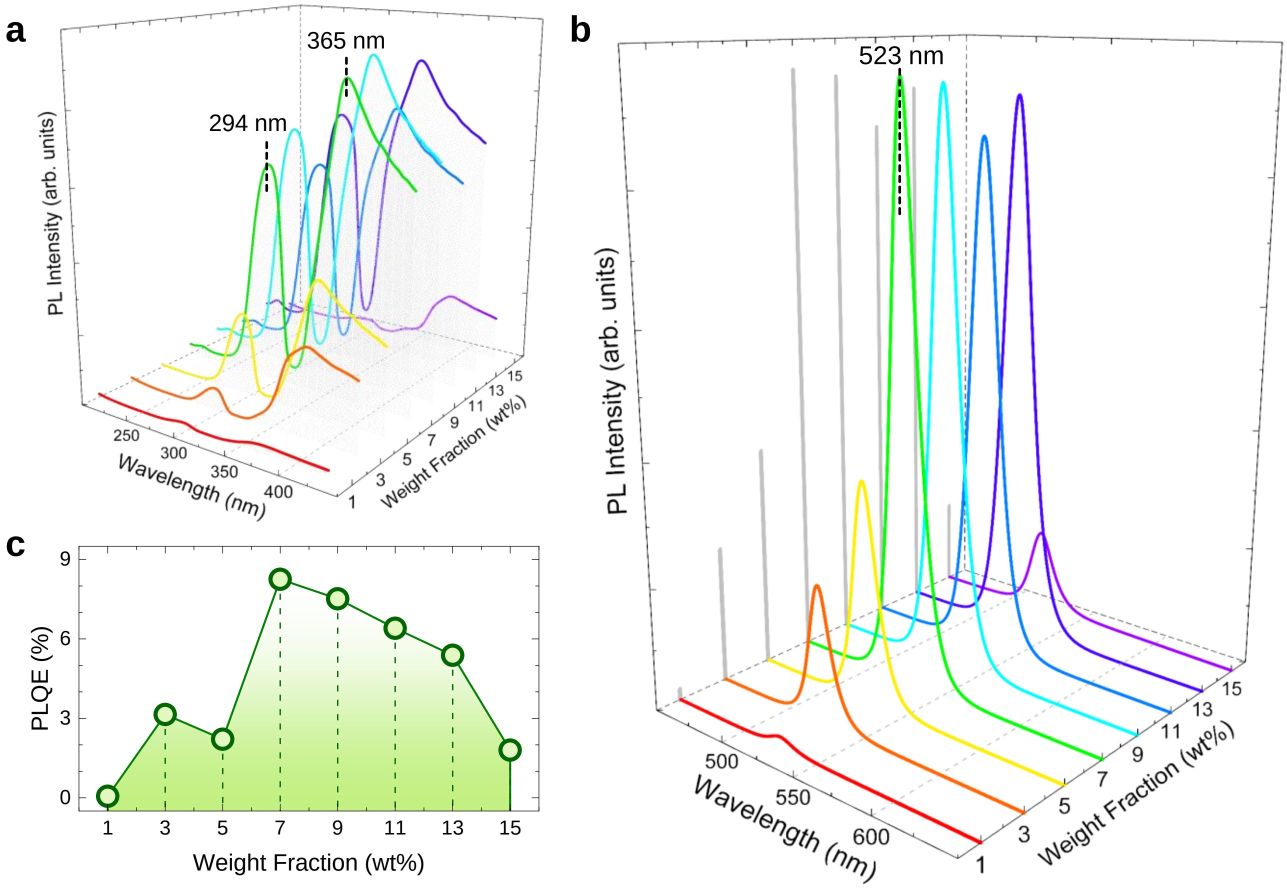

Supplementary Fig. 28 | Emissive properties of 0D/3D heterostructure. a, PLE spectra monitored at emission wavelength of 523 nm. Two peaks observed in each sample are located at 294 nm and 365 nm. **b,** PL spectra under excitation wavelength of 365 nm. Peak position of all samples is located at 523 nm. **c,** PLQE values with change in weight fraction of 3D $CsPbBr_3$ under excitation wavelength of 365 nm.

4.3 Structural and emissive properties of Cases 1, 2, and 3

Rietveld refinement process of the PXRD patterns from Cases-1, 2, and 3 samples is shown in Supplementary Fig. 29. All the samples were designed to have 7 wt% of 3D $CsPbBr_3$ according to Fig. 5a of the main text and the refinement results prove that the Cases-1, 2, and 3 samples contain 6.5, 6.2, and 6.4 wt%, respectively. Overall, the proportions of 3D $CsPbBr_3$ in all three cases are similar.

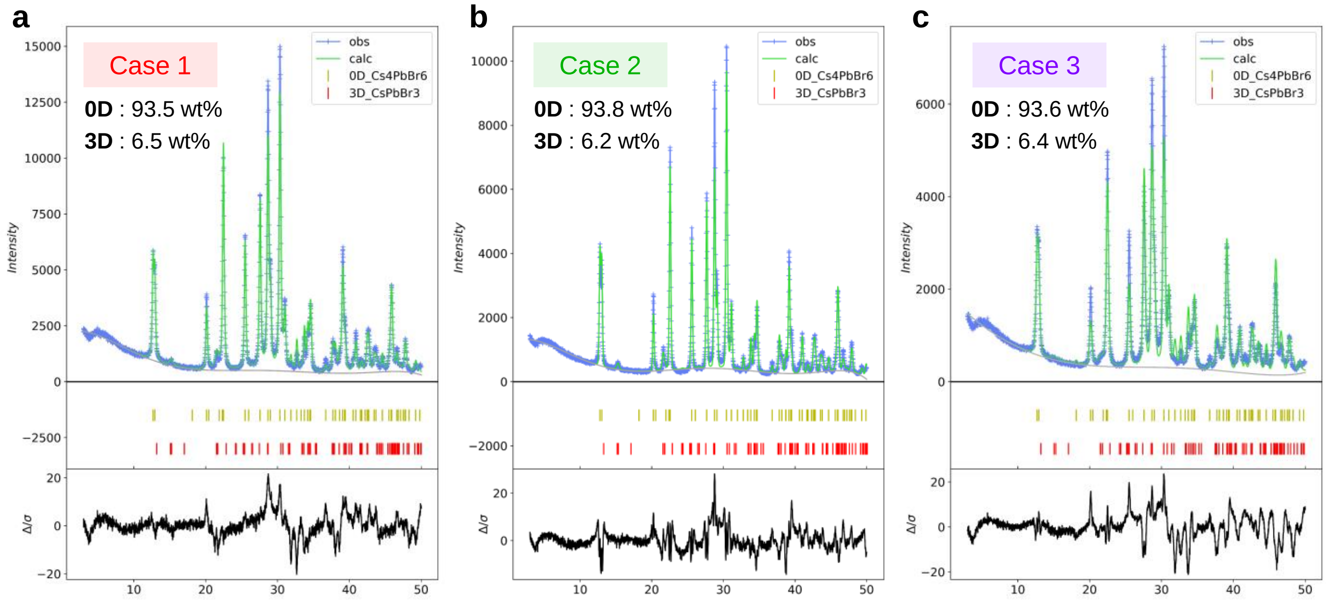

Supplementary Fig. 29 | Compositional ratio of Cases 1, 2, and 3 samples. a–c, Rietveld refinement results of Case-1 (**a**), Case-2 (**b**), and Case-3 samples (**c**).

Supplementary Figure 30 shows PLE and PL spectra of Case-1, Case-2, and Case-3 samples in powder form. In accordance with the trend in PLQE of the samples (Fig. 5b from the main text), Case-1 sample exhibits the highest PL intensity, Case-3 sample exhibits the least, and Case-2 sample is in between. Moreover, the “hole-burning” region between 294 nm and 365 nm regions in Supplementary Fig. 30a which is similar to that of Supplementary Fig. 7a, indicates the existence of direct interface between 0D Cs_4PbBr_6 and 3D $CsPbBr_3$.

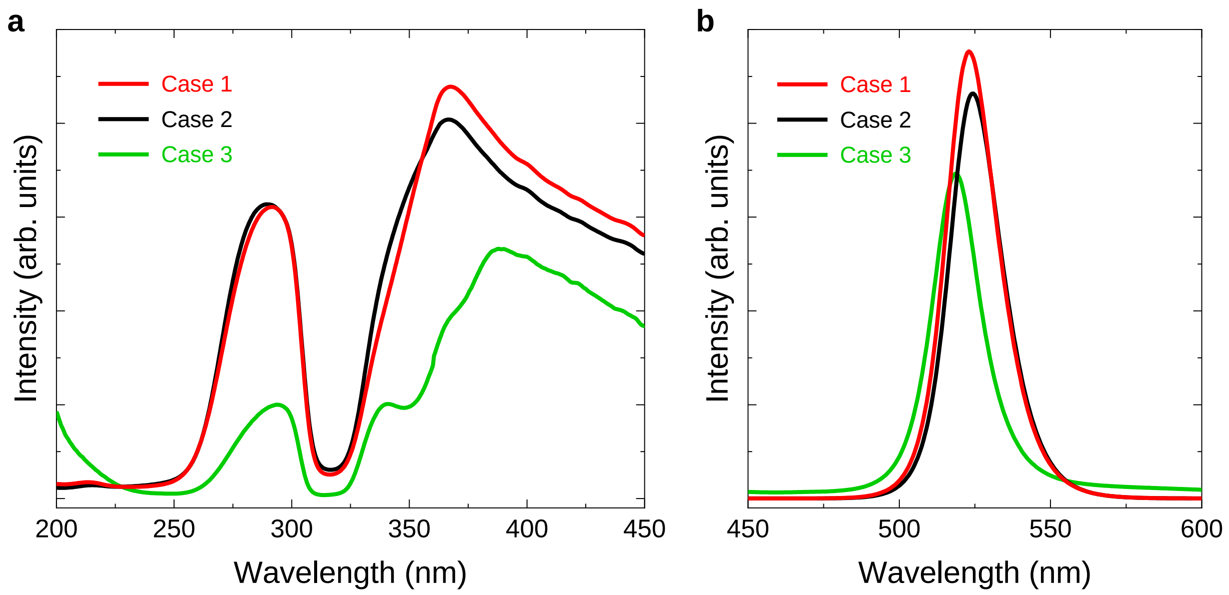

Supplementary Fig. 30 | Emissive properties of Cases 1, 2, and 3 samples. a,b, PLE spectra monitored at emission wavelength of 523 nm (**a**) and PL spectra with excitation wavelength of 365 nm (**b**) for Cases-1, 2, and 3 samples in poweder form.

4.4 ssNMR analyses of Cs_4PbBr_6/$CsPbBr_3$ hetero-structured powder samples

In order to identify any novel Cs envionments in the highly emissive MCS products, ssNMR analyses were conducted for Case-1 and Case-3 powder samples, and the full spectrum of each sample is shown in Supplementary Fig. 31a. Note that the spectrum of Cs_4PbBr_6 which is shown as a gray line is the same data from that of the 2880-min sample in Supplementary Fig. 19. Semi-transparent blue strips indicate peaks from Cs_4PbBr_6 with two Cs environments, Cs(1) and Cs(2), and semi-transparent yellow strip indicates peaks from $CsPbBr_3$. In accordance with the PXRD data from Supplementary Figs. 29a and 29c, signature of $CsPbBr_3$ is confirmed in the Case-1 and Case 3 samples.

Supplementary Figure 31b shows a closer view near the Cs(1) peak of 226.5 ppm where a shoulder peak in the lower shift region within the Cs(1) peak is identified for Case-1 and Case-3 samples (marked as semi-transparent purple strip). Moreover, the intensity of the shoulder peak is higher in the Case-1 sample than in the Case-3 sample; note that this result correlates with the emissivitiy of these samples (Fig. 5b of the main text) where Case-1 shows a higher PLQE than Case-3.

This shoulder-like feature was previously reported in ^{133}Cs spectra of Cs_4PbBr_6 single crystals[32], which was attributed to Cs atoms near 'superparamagnetic' bromine vacancies present in the structure. Thus, in an attempt to identify the nature of this peak, we measured the ssNMR spectrum of Case-1 sample at different recycle delays (d_{rcyc}); at 180 s and 0.2 s (left and right figures of Supplementary Fig. 31c, respectively). NMR resonances near paramagnetic environments typically experience much faster relaxation than their diamagnetic counterparts due to the electron-nuclear hyperfine coupling; therefore, if this additional resonance indeed arises from such paramagnetic defects, we would expect it to relax much faster than the native 0D resonances and consequently grow in intensity (relative to the slow-relaxing 0D peaks which would saturate) when d_{rcyc} is reduced to 0.2 s. Note that all of the spectra in Supplementary Figs. 31a and 31b were obtained under d_{rcyc} at 180 s. Each spectrum of Supplementary

Fig. 31c is deconvoluted into three gaussian functions and the fitting parameters are tabulated in Supplementary Table 1. An overlap of the third peaks from different d_{rcyc} settings is presented in the inset of Supplementary Fig. 31c, which importantly shows almost no difference in the intensity between the two spectra (d_{rcyc}=180 and 0.2 s). Therefore, unlike the previous study, we suspect that this new Cs resonance does not arise from the paramagnetic defects, but rather from a diamagnetic Cs environment with a structural similarity to that of the native 0D Cs local structure (small differences in chemical shift). While the exact nature of this shoulder peak is difficult to identify at this moment, we speculate that it may arise from the 0D Cs atoms residing in the interface between Cs_4PbBr_6 and $CsPbBr_3$, possibly experiencing a minor structural distortion to accommodate the (small) lattice mismatch between the two phases (~4.7 % between d_{100} of Cs_4PbBr_6 and $3d_{110}$ of $CsPbBr_3$ is reported[33]) which accounts for the small decrease in chemical shifts. Confirmation of this assignment is beyond the scope of this study and further work is being carried out.

The same interpretation is applied to the Case-3 sample, which is presented in Supplementary Fig. 31d with the fitting parameters tabulated in Supplementary Table 2. The inset of Supplementary Fig. 31d also shows negligible difference in the intensity between the shoulder peaks. Note that the experimental conditions for acquiring the ssNMR spectra for Case-1 and Case-3 samples are the same as that in the Methods section only without the use of Kel-F inserts.

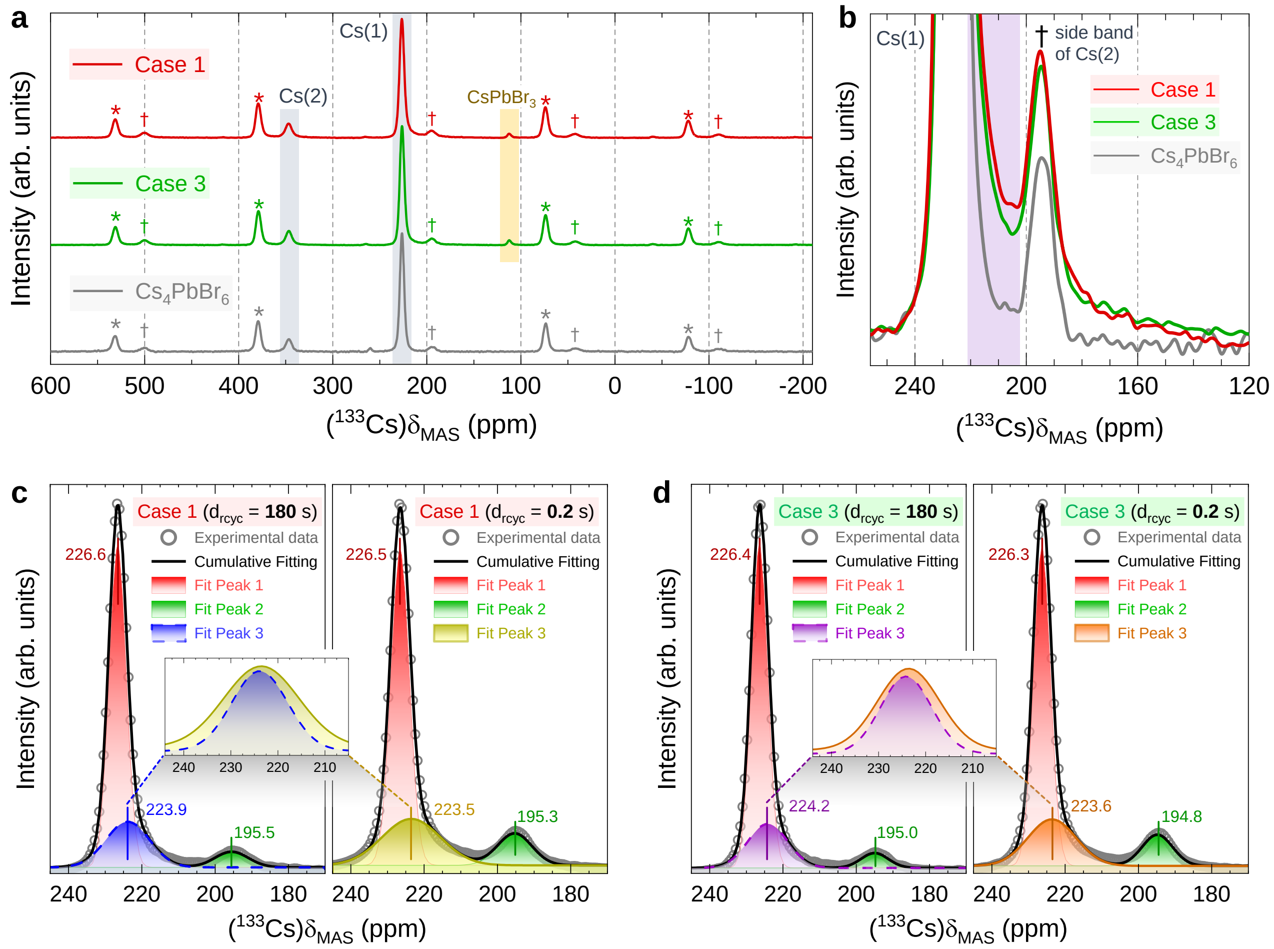

Supplementary Fig. 31 | ssNMR analyses for hetero-structured powder samples. a, ssNMR spectra of Case-1, Case-2, and Cs_4PbBr_6 powder samples. Semi-transparent blue strips are signs of ^{133}Cs at the local structure of Cs_4PbBr_6, and semi-transparent yellow strip is a sign of $CsPbBr_3$. The remaining peaks marked as asterisks (*) and daggers (†) are spinning side bands of Cs(1) peak and Cs(2) peak, respectively, which are all from Cs_4PbBr_6. **b,** Magnified view of **a** ranging from 120 ppm to 256 ppm. Semi-transparent purple strip indicates the existence of a shoulder peak attached to the Cs(1) peak for Case-1 and Case-3 samples. **c,d,** ssNMR of Case-1 (**c**) and Case-3 (**d**) samples at different recycle delays (d_{rcyc}) of 180 s (left) and 0.2 s (right). Both spectra are respectively deconvoluted into three gaussian functions, each filled with different colors.

Supplementary Table 1 | Fitting parameters of Supplementary Fig. 31c.

**Case 1** (ppm)	**d_{rcyc} = 180 s**			**d_{rcyc} = 0.2 s**		
	Peak 1	Peak 2	Peak 3	Peak 1	Peak 2	Peak 3
Peak Center	226.60	195.50	223.86	226.54	195.27	223.52
FWHM	5.54	10.81	13.98	5.95	11.02	18.76
Integration	511.05	48.37	183.43	537.44	100.83	250.01

Supplementary Table 2 | Fitting parameters of Supplementary Fig. 31d.

**Case 3** (ppm)	**d_{rcyc} = 180 s**			**d_{rcyc} = 0.2 s**		
	Peak 1	Peak 2	Peak 3	Peak 1	Peak 2	Peak 3
Peak Center	226.40	194.98	224.15	226.34	194.84	223.60
FWHM	5.28	8.76	12.71	5.48	9.82	15.45
Integration	489.68	37.46	161.24	499.75	87.94	206.84

4.5 HRTEM analyses of Cases 1, 2, and 3 powder samples

Supplementary Figure 32 shows transmission electron microscopy (TEM) along with high-resolution TEM (HRTEM) observation for Case-1 samples. Hexagonal-shaped crystals are seen in Supplementary Fig. 32a, wherein the part marked as dotted white square is presented in Supplementary Fig. 32b. Inset of Supplementary Fig. 32b shows the (300) lattice planes of 0D Cs_4PbBr_6.

Supplementary Figure 33 shows multiple detailed images under HRTEM observation for Case-1 sample. Similar to Fig. 5g of the main text, lattice patterns corresponding to 3D $CsPbBr_3$ grid were found inside the grain of the sample. This result is in accordance with the multiple $CsPbBr_3$ peaks detected in selected area electron diffraction (SAED) analysis (Figs. 4d and 4f of the main text). Note that when two grids of each lattice overlap, it is facile to distinguish whether the entity is 3D $CsPbBr_3$ or 0D Cs_4PbBr_6 because 3D $CsPbBr_3$ has orthorhombic structure which means that lattices overlap with an angle of 90 degrees.

Supplementary Figure 34a is an identical figure with the left image of Fig. 5g only without false colors. Supplementary Figures 34b–e shows magnified view of each square in Fig. 5g and their corresponding fast Fourier transformed (FFT) images. Note that $0D_I$ and $3D_I$ images were presented in Fig. 5g.

Supplementary Figure 35 shows multiple images under HRTEM observation for Case-3 samples. In contrast to Fig. 5g and Supplementary Fig. 33, lattice patterns corresponding to 3D $CsPbBr_3$ grid were not found. Only the lattice spacings (*d* spacings) of 0D Cs_4PbBr_6 were observed. Since synthesis through Case 3 induces 3D $CsPbBr_3$ to form on the surface or the exterior part of 0D Cs_4PbBr_6 host (see the illustration in the lower panel of Fig. 5c from the main text), it cannot be encapsulated inside the 0D matrix and therefore is directly exposed to high-energy electron beam with an acceleration volage of 200 kV. Its high susceptibility to such high energy electron beam compared to 0D counterpart[8,34] makes it

hardly detectable under HRTEM observation. This result is in accordance with the absence of $CsPbBr_3$ peaks in SAED analysis (Figs. 4e and 4f).

d spacings' reference of 3D $CsPbBr_3$ is from lattice parameters of a = 8.24 Å, b = 11.74 Å, c = 8.20 Å, α = β = γ= 90° in the Pnma space group[4] and 0D Cs_4PbBr_6 is from lattice parameters of a = b = 13.72 Å, c = 17.29 Å, α = β = 90°, γ = 120° in the R−3c space group[5]. Note that all of the TEM and HRTEM images presented in Supplementary Figs. 32–35 are powder samples of Cases 1–3 dispersed in toluene.

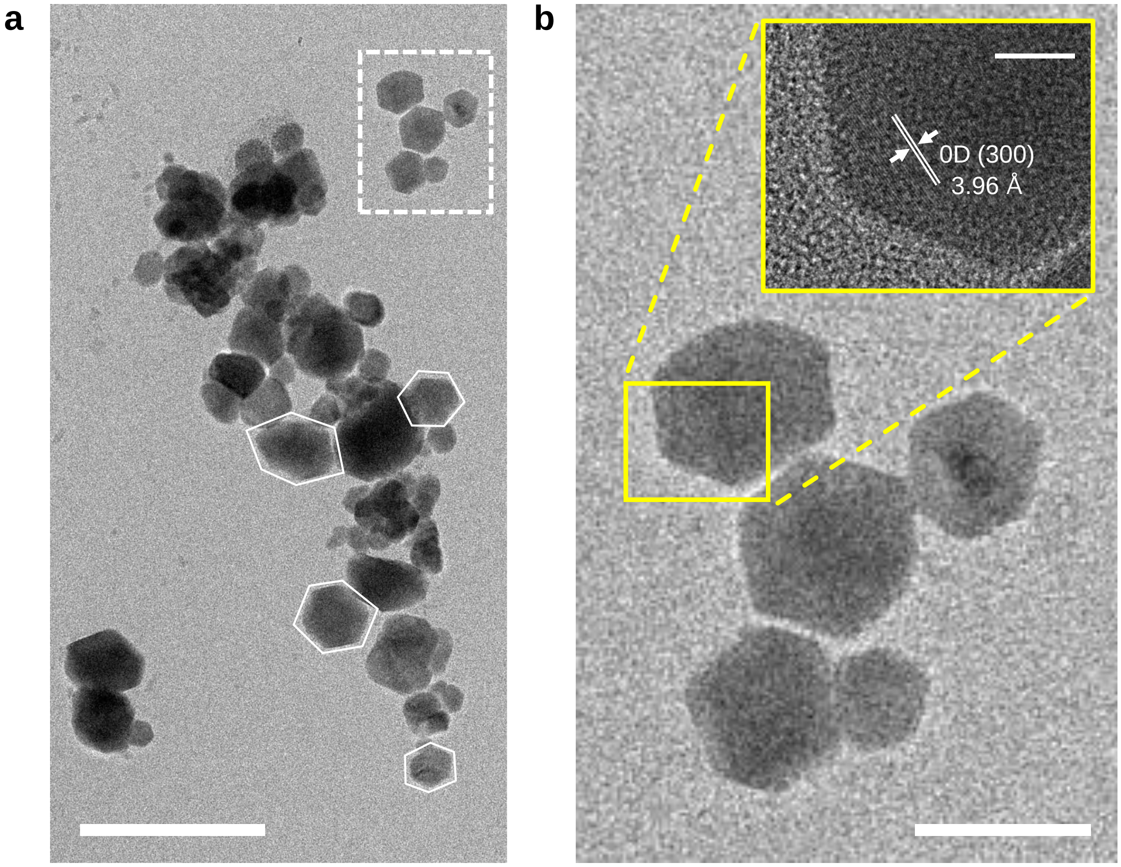

Supplementary Fig. 32 | TEM image of Case 1. a,b, TEM image of Case-1 samples (scale bar, 200 nm; **a**) and magnified view of the dotted white box in **a** (scale bar, 50 nm; **b**). Inset in **b** shows (300) lattice planes corresponding to 0D Cs_4PbBr_6 (scale bar, 10 nm).

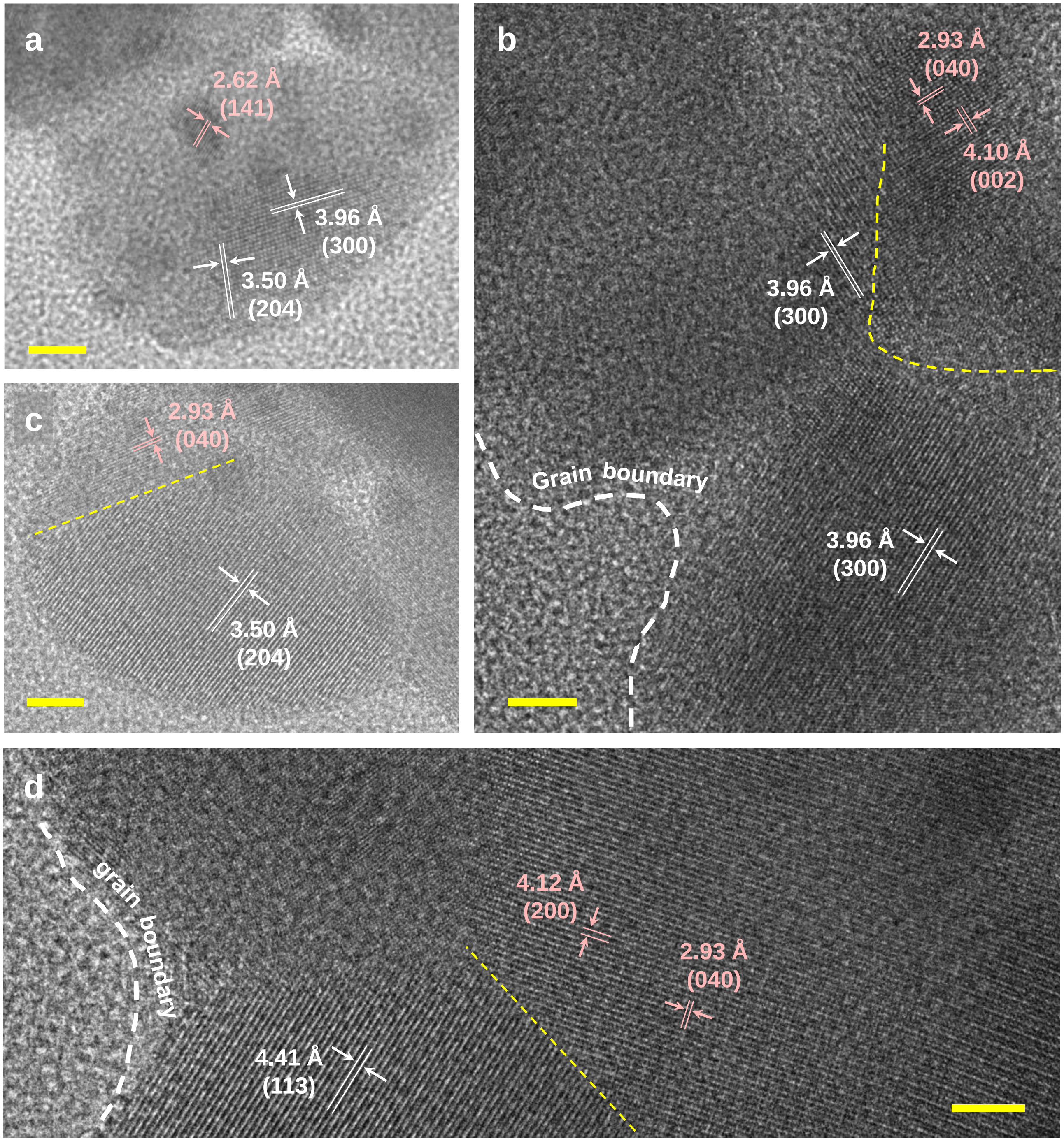

Supplementary Fig. 33 | HRTEM observation of Case-1 samples. a–d, *d* spacings and their corresponding lattice planes for 0D Cs_4PbBr_6 lattices (written in white) and for 3D $CsPbBr_3$ lattices (written in pink). All yellow scale bars, 5 nm.

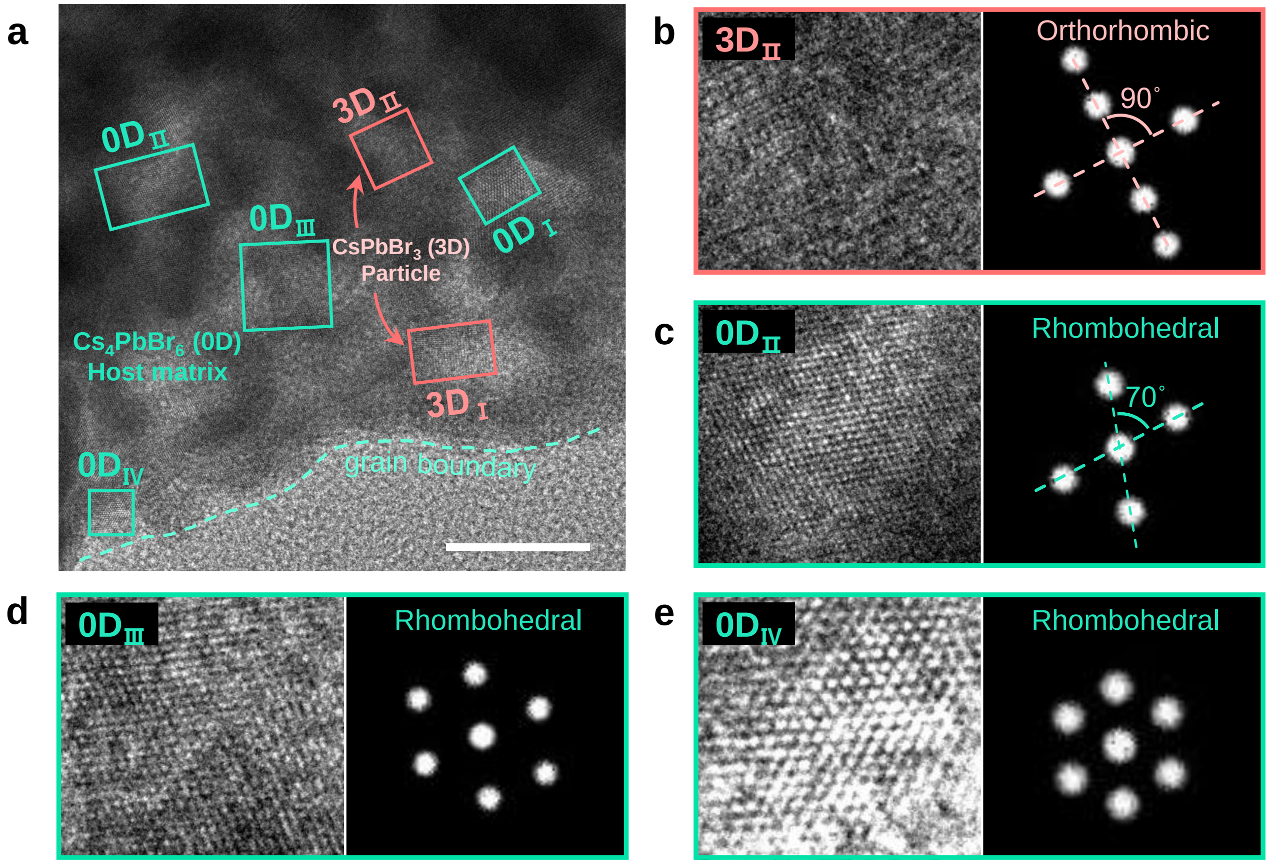

Supplementary Fig. 34 | Mixture of 0D and 3D phases in Case 1. a, Same figure from left image of Fig. 5g in the main text only without false colors. Scale bar, 20 nm. **b–e,** Magnified view of the squares in **a** (left) and their corresponding FFT images (right).

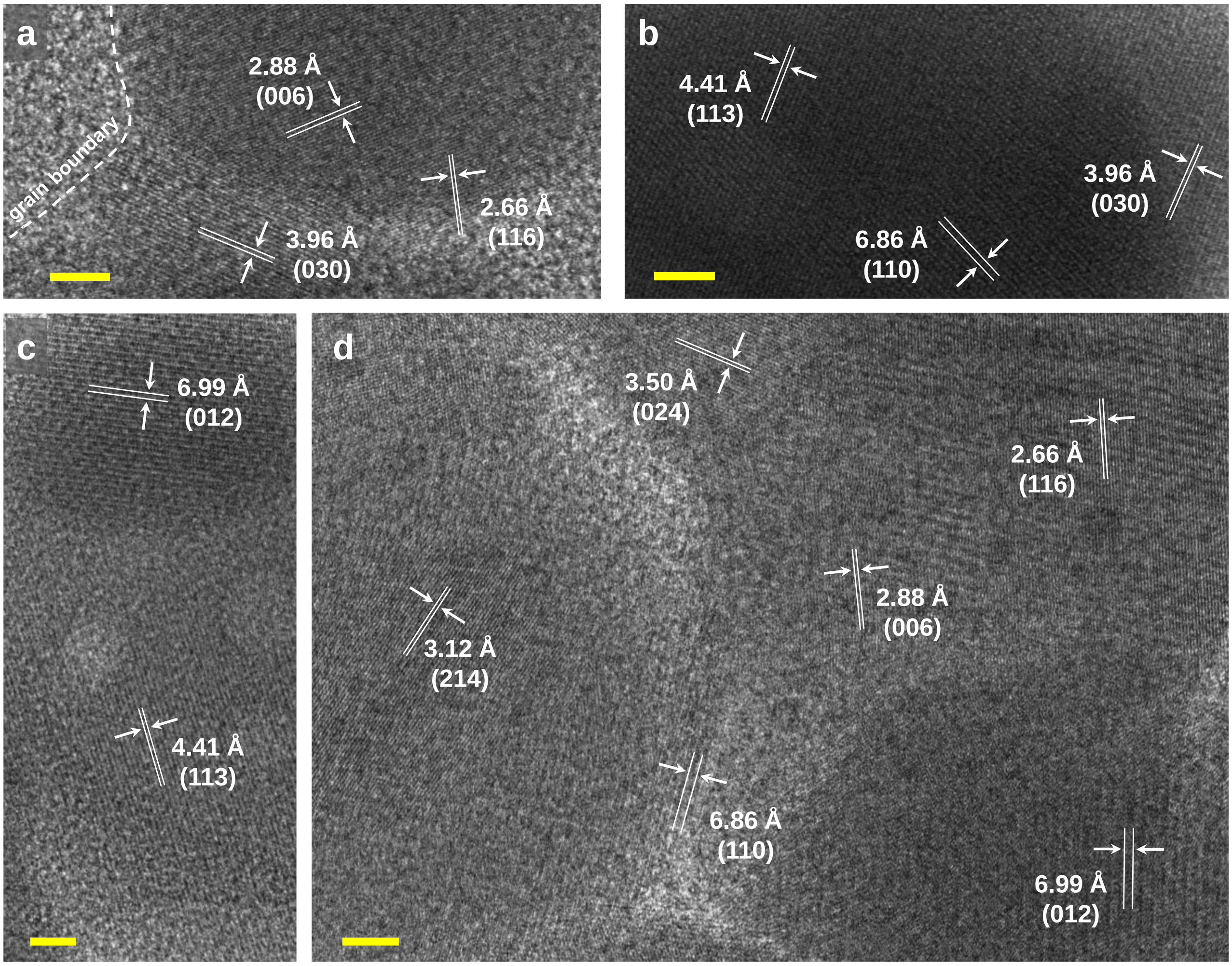

Supplementary Fig. 35 | HRTEM observation of Case-3 samples. a–d, All *d* spacings and their corresponding lattice planes for 0D Cs_4PbBr_6 lattices. All yellow scale bars, 5 nm.

4.6 Ligand-capped NCs through wet ball milling

Supplementary Figures 36a–c shows the PL spectra of ligand-capped Cases-1, 2, and 3 nanocrystals (NCs), respectively, with an excitation wavelength of 405 nm. Supplementary Figure 36d shows the absorption spectra of ligand-capped Cases-1, 2, and 3 NCs where we can observe two peaks located at 310 nm and 360 nm. A magnified view near the 500 nm is presented in the inset and we can also observe the band-to-band absorption with an onset of ~525 nm. The band-to-band absorption is attributed to the small amount of 3D $CsPbBr_3$ contained within the samples.

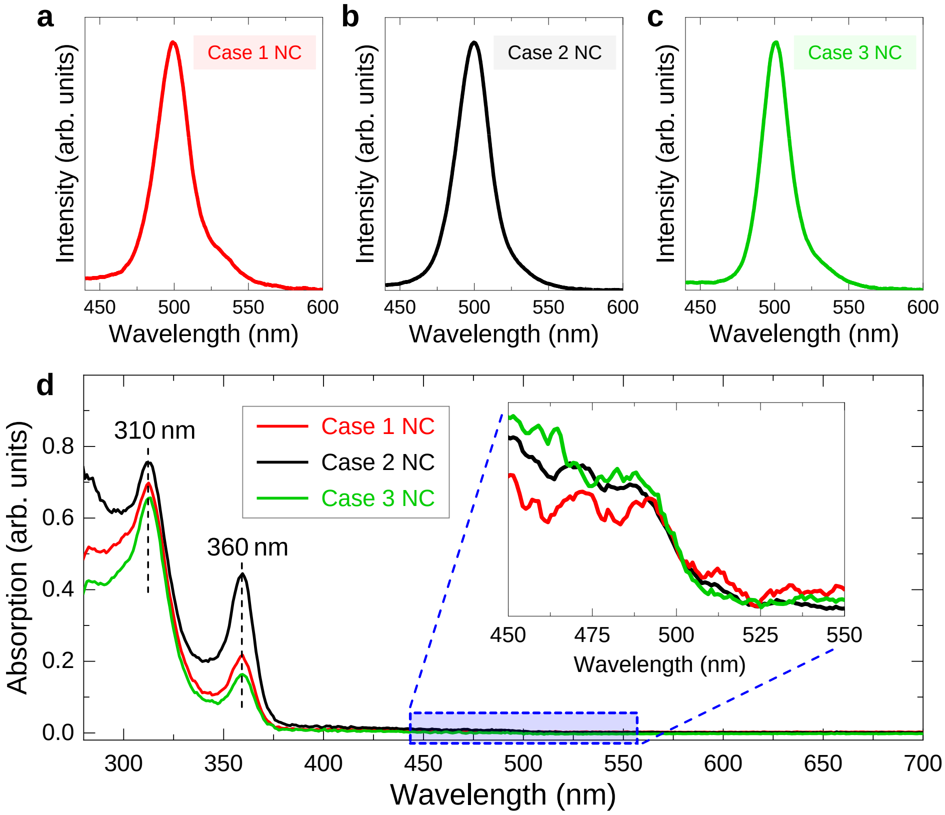

Supplementary Fig. 36 | Optical properties of ligand-capped NCs. a–c, PL spectrum of Case 1 (**a**), Case 2 (**b**), and Case 3 NCs (**c**) with an excitation wavelength of 405 nm. **d**, Absorption spectrum of Cases 1, 2, and 3 NCs.

4.7 Contour plots of PL intensity as a function of excitation and emission wavelengths

Supplementary Figures 37 and 38 shows contour plots of PL intensity as a function of excitation and emission wavelengths from each corresponding sample. Excitation wavelength range from 245 to 450 nm and emission wavelength range from 250 to 700 nm. Note that the color gradients in all figures are presented in logarithmic scales. We can identify that Fig. 5h of the main text can be similarly reconstructed if one simply performs a linear combination of the spectra of 0D and 3D NCs, as shown in Supplementary Figs. 37a and 37b, respectively. This further proves that the spectra shown in Fig. 5h represent the spectra for a simple mixture of 0D and 3D NCs, without any unintentional reactions between the two different components. Moreover, the PL quenching at the excitation wavelength of ~320 nm (area shown in the inset of Fig. 5i from the main text) is also observed in Case-2 NC (Supplementary Fig. 37c) and Case-3 NC (Supplementary Fig. 37d) samples similarly to Case-1 NC shown in Fig. 5i.

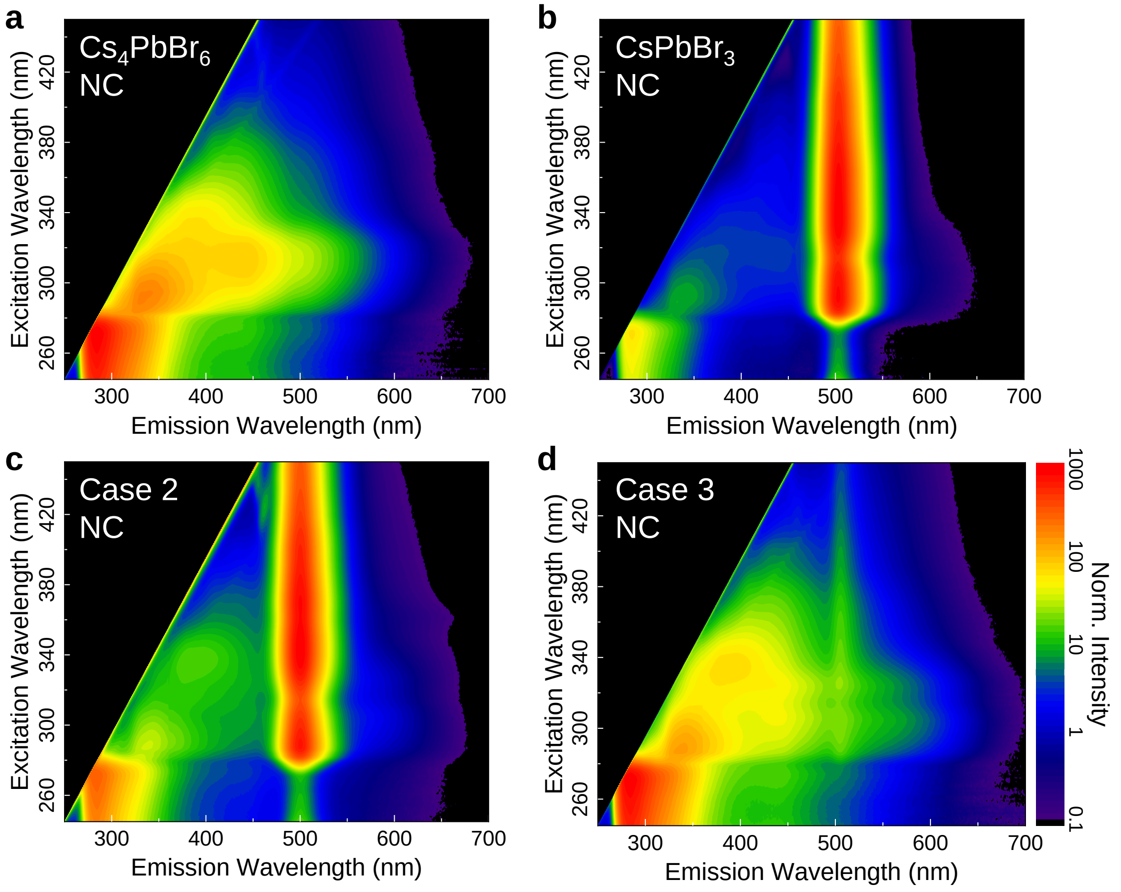

Supplementary Fig. 37 | Emission of Cases 1, 2, and 3 in the form of nanocrystals. a–d, Contour plots of PL intensity from Cs_4PbBr_6 NC (**a**), $CsPbBr_3$ NC (**b**), Case-2 NC (**c**), and Case-3 NC (**d**). Color gradient scales in **a–c** follows that of the scale presented in **d**.

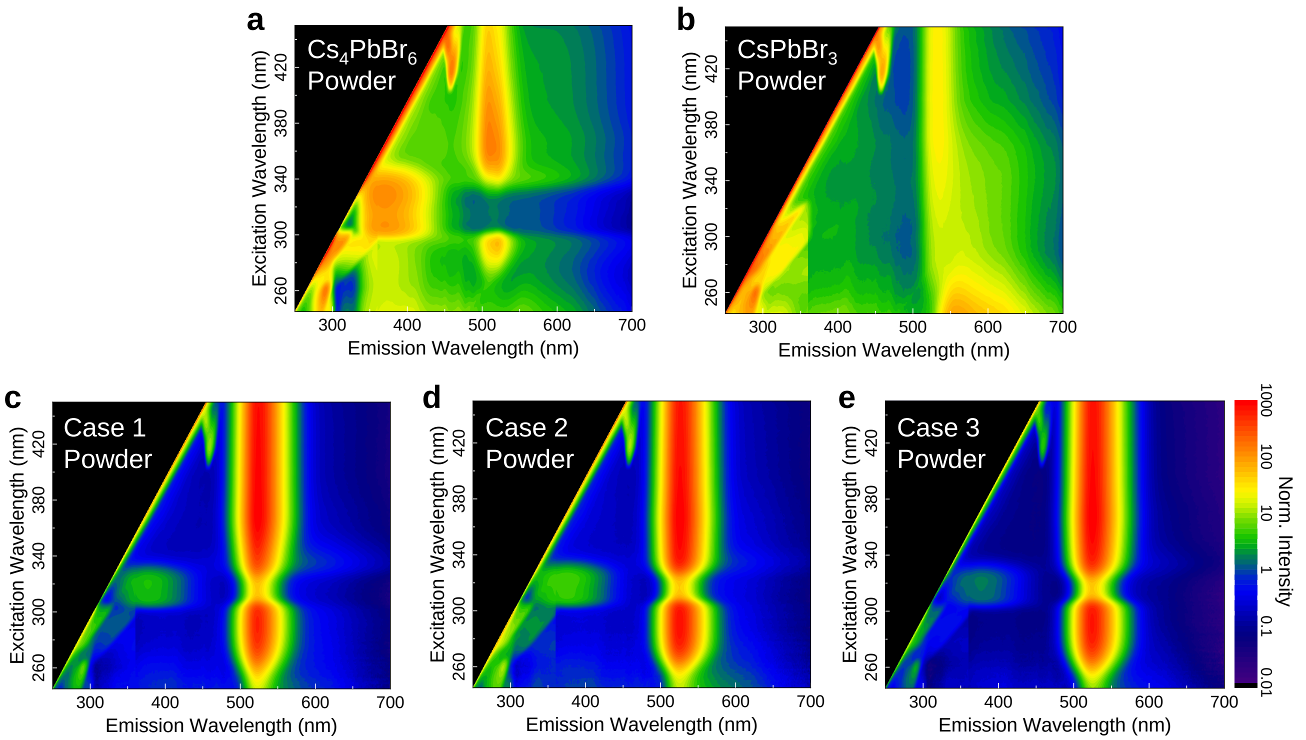

Supplementary Fig. 38 | Emission of 0D, 3D, and Cases 1, 2, and 3 in the form of powder. a–e, Contour plots of PL intensity from Cs_4PbBr_6 (**a**), $CsPbBr_3$ (**b**), Case-1 (**c**), Case-2 (**d**), and Case-3 (**e**) powder. Color gradient scales in **a–d** follows that of the scale presented in **e**.

Supplementary References

1. Udayabhaskararao, T. et al. A mechanistic study of phase transformation in perovskite nanocrystals driven by ligand passivation. *Chem. Mater.* **30**, 84–93 (2018).
2. Wu, L. et al. From nonluminescent Cs_4PbX_6 (X = Cl, Br, I) nanocrystals to highly luminescent $CsPbX_3$ nanocrystals: Water-triggered transformation through a CsX-stripping mechanism. *Nano Lett.* **17**, 5799–5804 (2017).
3. Yang, H. et al. Room-temperature engineering of all-inorganic perovskite nanocrsytals with different dimensionalities. *Chem. Mater.* **29**, 8978–8982 (2017).
4. Stoumpos, C. C. et al. Crystal growth of the perovskite semiconductor $CsPbBr_3$: A new material for high-energy radiation detection. *Cryst. Growth Des.* **13**, 2722–2727 (2013).
5. De Bastiani, M. et al. Inside perovskites: Quantum luminescence from bulk Cs_4PbBr_6 single crystals. *Chem. Mater.* **29**, 7108–7113 (2017).
6. Zhang, Z. et al. Growth, characterization and optoelectronic applications of pure-phase large-area $CsPb_2Br_5$ flake single crystals. *J. Mater. Chem. C* **6**, 446–451 (2018).
7. de Mello, J. C., Wittmann, H. F. & Friend, R. H. An improved experimental determination of external photoluminescence quantum efficiency. *Adv. Mater.* **9**, 230–232 (1997).
8. Saidaminov, M. I. et al. Pure Cs_4PbBr_6: Highly luminescent zero-dimensional perovskite solids. *ACS Energy Lett.* **1**, 840–845 (2016).
9. Chen, Y.-M. et al. $Cs_4PbBr_6/CsPbBr_3$ perovskite composites with near-unity luminescence quantum yield: Large-scale synthesis, luminescence and formation mechanism, and white light-emitting diode application. *ACS Appl. Mater. Interfaces* **10**, 15905–15912 (2018).
10. Cao, F. et al. Shining emitter in a stable host: Design of halide perovskite scintillators for X-ray imaging from commercial concept. *ACS Nano* **14**, 5183–5193 (2020).

11. Cao, F. et al. $CsPbBr_3@Cs_4PbBr_6$ emitter-in-host composite: Fluorescence origin and interphase energy transfer. *J. Phys. Chem. C* **125**, 3–19 (2020).

12. Nikl, M. et al. Photoluminescence of Cs_4PbBr_6 crystals and thin films. *Chem. Phys. Lett.* **306**, 280–284 (1999).

13. Kondo, S. et al. Localized optical absorption in Cs_4PbBr_6. *J. Phys. Condens. Matter* **14**, 2093–2099 (2002).

14. Yin, J. et al. Intrinsic lead ion emissions in zero-dimensional Cs_4PbBr_6 nanocrystals. *ACS Energy Lett.* **2**, 2805–2811 (2017).

15. Wang, Y. et al. Solution-grown $CsPbBr_3/Cs_4PbBr_6$ perovskite nanocomposites: Toward temperature-insensitive optical gain. *Small* **13**, 1701587 (2017).

16. Akkerman, Q. A. et al. Zero-dimensional cesium lead halides: History, properties, and challenges. *J. Phys. Chem. Lett.* **9**, 2326–2337 (2018).

17. Zhang, Z. et al. Aqueous solution growth of millimeter-sized nongreen-luminescent wide bandgap Cs_4PbBr_6 bulk crystal. *Cryst. Growth Des.* **18**, 6393–6398 (2018).

18. Jacobs, P. W. M. Alkali halide crystals containing impurity ions with the ns^2 ground-state electronic configuration. *J. Phys. Chem. Solids* **52**, 35–67 (1991).

19. Newbury, D. E. et al. Is scanning electron microscopy/energy dispersive x-ray spectrometry (SEM/EDS) quantitative? *Scanning* **35**, 141–168 (2013).

20. Chen, D. et al. Large-scale room-temperature synthesis and optical properties of perovskite-related Cs_4PbBr_6 fluorophores. *J. Mater. Chem. C* **4**, 10646–10653 (2016).

21. Zhang, Y. et al. Zero-dimensional Cs_4PbBr_6 perovskite nanocrystals. *J. Phys. Chem. Lett.* **8**, 961–965 (2017).

22. Snyder, R. et al. *Introduction to X-ray powder diffractometry*. p.315 (Wiley-Interscience, 1996).

23. Qin, Z. et al. Revealing the origin of luminescence center in 0D Cs_4PbBr_6 perovskite. *Chem. Mater.*

31, 9098–9104 (2019).

24. Cha, J.-H. et al. Photoresponse of $CsPbBr_3$ and Cs_4PbBr_6 perovskite single crystals. *J. Phys. Chem. Lett.* **8**, 565–570 (2017).
25. Calistru, D. M. et al. Identification of the symmetry of phonon modes in $CsPbCl_3$ in phase IV by Raman and resonance-Raman scattering. *J. Appl. Phys.* **82**, 5391–5395 (1997).
26. Yaffe, O. et al. Local polar fluctuations in lead halide perovskite crystals. *Phys. Rev. Lett.* **118**, 136001 (2017).
27. Dracopoulos, V. et al. Raman spectra and structure of $PbCl_2$–ACl (A=K,Cs) melts. *Polyhedron* **24**, 619–625 (2005).
28. Jung, Y.-K. et al. Intrinsic doping limit and defect-assisted luminescence in Cs_4PbBr_6. *J. Mater. Chem. A* **7**, 20254–20261 (2019).
29. Yin, J. et al. Point defects and green emission in zero-dimensional perovskites. *J. Phys. Chem. Lett.* **9**, 5490–5495 (2018).
30. Du, P. et al. Efficient and large-area all vacuum-deposited perovskite light-emitting diodes via spatial confinement. *Nat. Commun.* **12**, 4751 (2021).
31. Cola, M. et al. Binary systems formed by lead bromide with (Li, Na, K, Rb, Cs and Tl)Br: a DTA and diffractometric study. *Z. Naturforsch., A: Phys. Sci.* **26**, 1328–1332 (1971).
32. Cha, J.-H. et al. Superparamagnetism of green emissive Cs_4PbBr_6 zero-dimensional perovskite crystals. *ACS Energy Lett.* **5**, 2208–2215 (2020).
33. Bao, Z. et al. Efficient luminescence from $CsPbBr_3$ nanoparticles embedded in Cs_4PbBr_6. *J. Phys. Chem. Lett.* **11**, 7637–7642 (2020).
34. Zhang, D. et al. Atomic-resolution transmission electron microscopy of electron beam–sensitive crystalline materials. *Science* **359**, 675–679 (2018).
